# Reversible 2D Supramolecular Organic Frameworks encompassing Viologen Cation Radicals and CB[8]

**DOI:** 10.1038/s41598-018-19739-7

**Published:** 2018-01-22

**Authors:** Kanagaraj Madasamy, Vellaiah Maruthiah Shanmugam, David Velayutham, Murugavel Kathiresan

**Affiliations:** 1 Electroorganic Division, CSIR-Central Electrochemical Research Institute, Karaikudi, 630003 Tamil Nadu India; 2 Academy of Scientific and Innovative Research (AcSIR), CSIR-Central Electrochemical Research Institute, Karaikudi, 630003 Tamil Nadu India

## Abstract

Reversible 2D supramolecular organic frameworks encompassing branched viologen architectures and cucurbit[8]uril (CB[8]) were constructed and investigated. UV-vis investigation clearly indicates the formation and intermolecular dimerization of monocation radicals and their encapsulation into the hydrophobic CB[8] cavity which is further complemented by EPR (electron paramagnetic resonance) spectroscopy. Particle size measurements by dynamic light scattering method showed particle sizes in the range of several µm indicating larger aggregates. Zeta potential measurements suggested the instability of these particles and their tendency to form aggregates. TEM (transmission electron microscope) analysis further revealed the formation of supramolecular polymer (monocation radical with cucurbit[8]uril) whose diameter were in the range of several µm as indicated by DLS measurements; however the oxidized form, i.e., the viologen dication with cucurbit[8]uril showed dotted spots in the range of sub nanometer level. The internal periodicities of the supramolecular polymers were analyzed by SAXs (small angle X-ray scattering) measurements. Additionally, we have demonstrated that these supramolecular organic frameworks can be depolymerized by oxidation in air and again can be polymerized (intermolecular radical dimerization) by reduction under inert atmosphere demonstrating that these systems will be of broad interest.

## Introduction

Supramolecular assembly encompasses molecular self-assembled structures, supramolecular polymers, molecular daisy chains, etc. that are governed through non-covalent interactions such as hydrogen bonding, hydrophobic interactions, electrostatic interactions, π-π interactions, etc^1^. They typically involve a host and a guest molecule and based on their presence in a chemical entity, the interactions can be either intramolecular or intermolecular. Most of these interactions are concentration dependent and are usually probed using techniques such as NMR [nuclear magnetic resonance techniques such as ^1^H NMR, DOSY (diffusion ordered spectroscopy)], cyclic voltammetry, UV-Vis, ITC (isothermal calorimetry), etc. These as formed supramolecular ensembles^2^ usually respond to external stimulus such as change in electrochemical potential, temperature, pH, solvent composition and light, etc^3^. Rational design and synthesis allows the preparation of variety of supramolecular polymers^4–8^, cross-linked gel materials^9,10^, hierarchical assemblies^11^ and metastable polymeric nanoparticles. In the last two decades, several supramolecular structures were designed by chemists using the concept of self-assembly^12^, templating and self-sorting strategies^13,14^. Complex-supramolecular architectures between electron deficient and electron rich moieties through non-covalent interactions were developed and were subjected to encapsulation upon addition of respective macrocyclic hosts such as crown ethers, cyclodextrins^15,16^, calixarenes^17^, cucurbiturils^18^ and pillararenes^19^. Cucurbit[n]uril (CB[n]), a condensed macrocyclic host molecule with n glyco-uril and n formaldehyde units have the ability to encapsulate different cationic and neutral guest molecules in aqueous medium^20^. Several studies were reported on the stable ternary inclusion complex formation between CB[8] host and viologen/different guests in different stoichiometric ratio^21^. Based on this unique property, CB[8] has been effectively employed as a linker in supramolecular assembly like supramolecular polymers, micelles, and hydrogels^22–25^. Recently some smart designed supramolecular polymers have been published by applying the strategy of homodimerization^26–29^ where chemically one electron reduced viologen species (V^+•^) undergo irreversible dimerization (V^+•^…^•+^V) and these dimerized species are subsequently included into CB[8] cavity via hydrophobic interactions. Gao *et al*. reported that 1,1′-binaphthyl inter-connected viologen based tweezer like molecules undergoes reversible modulation upon chemical or photochemical reduction^29,30^. Spectroelectrochemical investigations of different conjugated oligomeric viologens were reported by Zhao and coworkers. The authors illustrated that the viologen radical cations dimerized to linear supramolecular polymers^31^. Zhang *et al*. reported a two-dimensional viologen based supramolecular assembly formed by viologen radical dimerization which is enhanced by CB[8]^27^. Zhou *et al*. reported the construction of a three-dimensional viologen based cross-linked supramolecular assembly and they further discussed on the stability of radical dimer with preorganization of rigid building blocks^28^. Very recently Marchini *et al*. reported the pimerization of tetrahedrally arranged bipyridinium units wherein, reduced viologen units were intermolecularly dimerized, which stabilizedthe system as well asenchanced the supramolecular growth^32^. Following a similar strategy, herein we report the synthesis of star-shaped ethyl terminated viologen (**ESV**) and newly self-assembled network of different viologens (**EV**, **EDV**, **ETV** & **ESV**) driven by radical–radical (V^+•^…^•+^V) dimerization. Further these dimerized species forms a supramolecular polymeric network in the presence of CB[8] and these assemblies were subjected to spectroscopic investigations.

**Figure 1:**
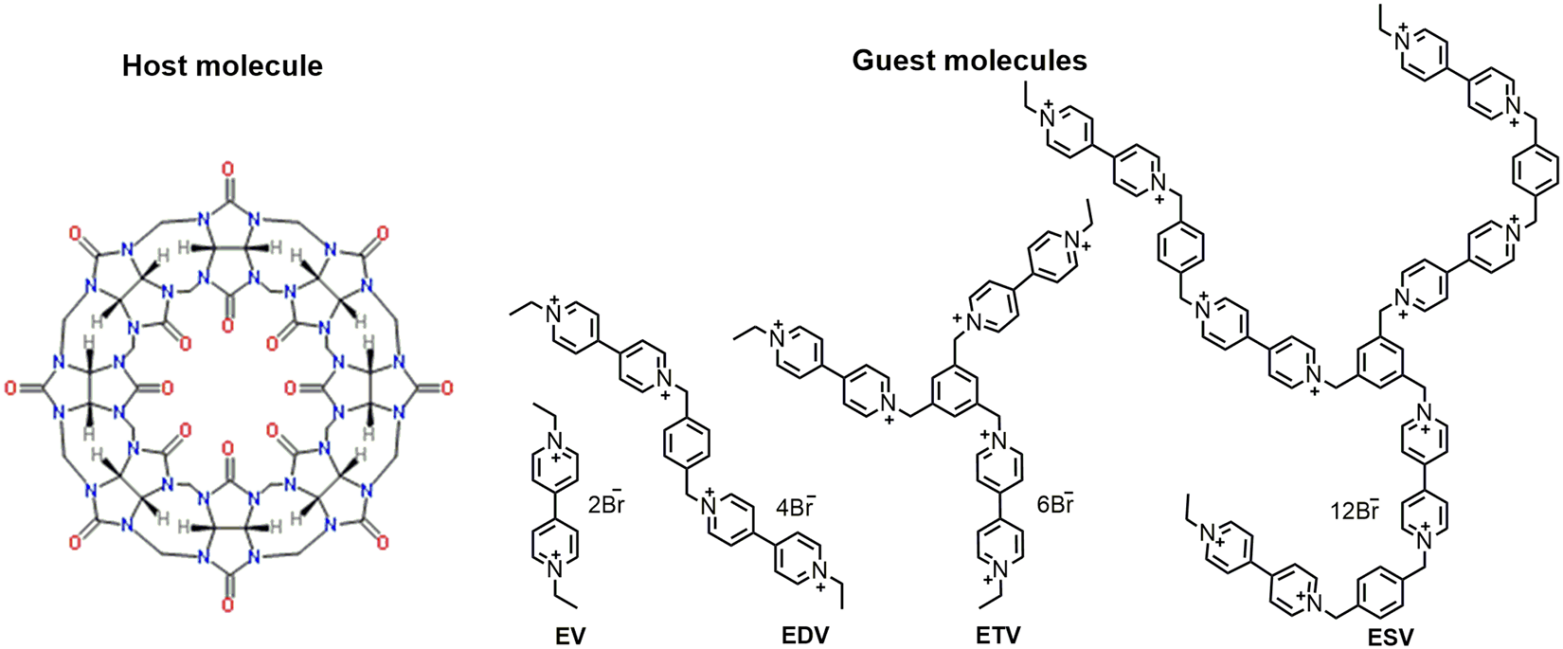
Structure of cucurbit[8]uril (CB[8]) host and viologen guest molecules **(EV**, **EDV**, **ETV** & **ESV**).

Figure 1 shows the chemical structure of cucurbit[8]uril host and viologen guest molecules.

## Results and Discussion

### Synthesis

**Figure 2:**
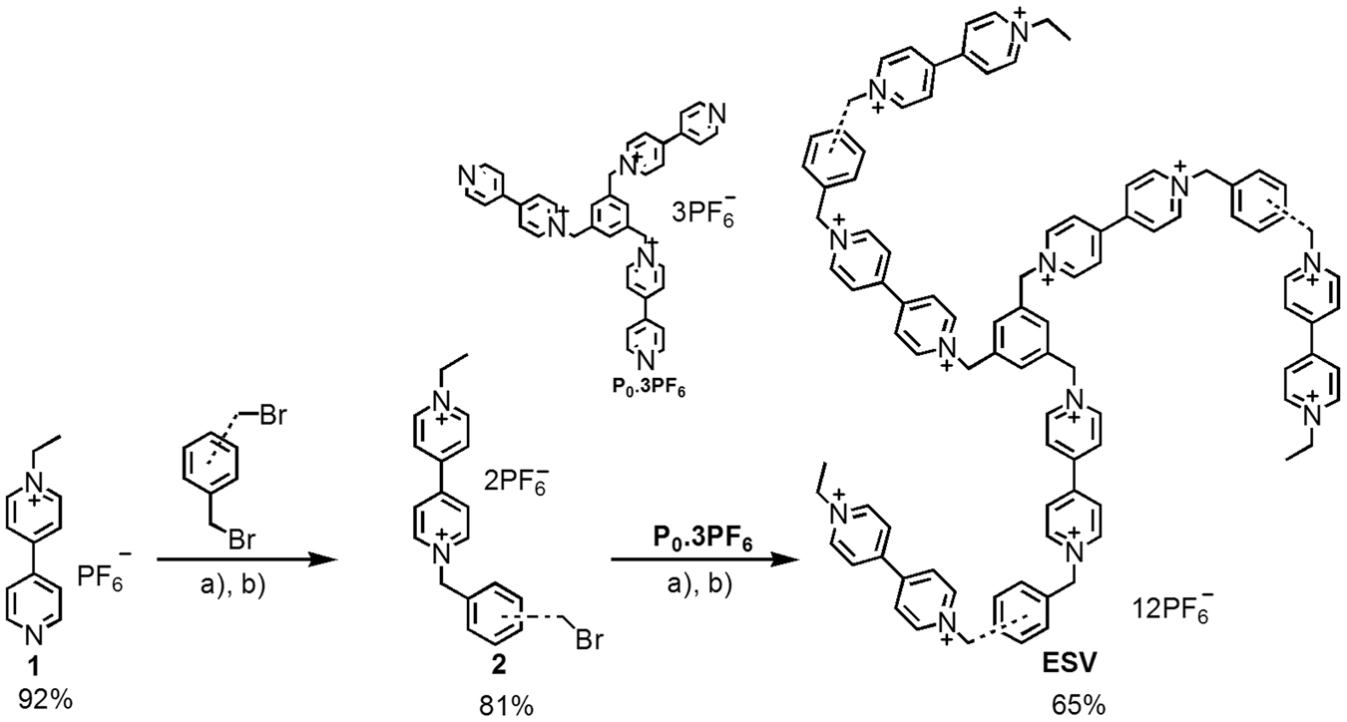
Synthesis of star shaped-viologen (**ESV)**: Reaction conditions; a) CH_3_CN, 80 °C, 2 d; b) 3 M NH_4_PF_6_/H_2_O.

Ethyl terminated star-shaped viologen (**ESV**) was synthesized in two steps following a reported procedure as shown in Fig. 2 ^16^. The synthesis starts with the selective alkylation reaction of 4,4′-bipyridine with 1-bromoethane to obtain the key compound **1** (92%) (Fig. 2)^17,33^. The compound **1** was further reacted with *p*-xylylene dibromide (five-fold excess) to obtain compound **2** (81%) in the first step. In the second step, compound **2** was reacted with well-known trimer core **P**_**0**_ **3PF**_**6**_^34^ to afford **ESV**(65%). The newly synthesized star-shaped viologen was characterized by ^1^HNMR, ^13^CNMR and DEPT measurements and its purity was cross-checked by elemental analysis.

## Methods

### General

All starting materials and solvents were purchased from Sigma-Aldrich or Alfa Aesar and used without further purification. All reactions were performed under dry conditions unless otherwise stated. Diethyl viologen dibromide was synthesized following the reported procedure and it was anion exchanged to **EV 2PF**_**6**_^35^. Cucurbit[8]uril was synthesized following a reported procedure. For measurements in organic media, the as prepared hexafluorophosphate salts were used, and for the measurements in aqueous media, the corresponding PF_6_¯ salts were anion exchanged to Br¯ salts by adding TBA.Br in CH_3_CN.

### Characterization and Measurements

Absorbance changes were measured with a Hewlett–Packard 8453 spectrophotometer. Electron Paramagnetic Resonance (EPR) parameters for all experiments are; Model = Bruker EMX Plus, Modulation amplitude = 4.0 G, Modulation frequency = 100 KHz, Centre Field = 3480, Sweep Width = 1000 G and Conversion time = 15 ms. Zeta potential and size distribution measurements were carried out in Dynamic Light scattering (DLS) method using Nano ZS model ZEN 3600, Malvern Instrument and Microtrac-Nanotrac 250 instrument respectively. ^1^H NMR, ^13^C NMR, and DEPT spectra were recorded on a Bruker 400 Avance spectrometer at 25 °C using CD_3_CN as a solvent and internal reference. Elemental analyses and cyclic voltammetric studies were performed on Elementar Vario EL III Micro cube instrument and CH- instrument respectively.

### UV-Vis, EPR and Electrochemistry

Absorbance and Electron Paramagnetic Resonance measurements were carried out in sodium phosphate buffer solution (0.1 M) containing sodium dithionite (50 mM) at RT. Cyclic voltammetry was carried out at room temperature in a standard three-electrode cell in aqueous medium. The electrolyte used was 0.1 M KCl/H_2_O. The working electrode was a glassy carbon disk (area = 0.07 cm^2^, Sinsil) and the counter electrode was a Pt wire. The reference electrode was Ag/AgCl/KCl (3 M) electrode (Sinsil) separated by a salt bridge containing 0.1 M KCl/H_2_O. Prior to each experiment, the working electrode was carefully polished with alumina powder and rinsed with distilled water. Potentiostatic control was provided by CH-Instrument and formal potentials (E^0′^) were calculated from cathodic and anodic peak potentials in CV’s according to E^0′^ = (E_pc_ + E_pa_)/2. The scan rate was 0.1 V/s.

### Zeta potential and particle size measurements

The size distribution of the viologen radical dimerized species in the presence and absence of **CB[8]** macro cyclic host molecule. For these analyses (0.1 M) sodium phosphate buffer solution containing (50 mM) sodium dithionite was used.

### Morphological analysis

The morphological structures of the prepared samples were captured using Scanning Electron Microscope (SEM) of TESCAN, VEGA 3 with Bruker Detecter, Zeck Republic. Transmission Electron Microscope (TEM) (Tecnai 20 G2 (FEI make), Netherlands, was used to analyze the surface morphology.

Small angle X-ray scattering experiments were conducted on Rigaku (Rigaku Smart Lab) using Cu Kα radiation, the power of X-ray source was operated at 50 mA and 40 kV and the d-spacing values were calculated by the formula d = 2π/q. For SAXs measurement, thin film of the respective polymer solution was dip coated on a glass plate and dried in glove box. The as dried thin films were then measured.

### Synthesis of Cucurbit[8]uril [CB[8]]

Reported procedure was followed to obtain **CB[8]**^36^ (17%).^1^H NMR(500 MHz, D_2_O): δ, ppm 5.80 (d, *J* = 15 Hz, 2H), 5.52 (s, 2H), 4.20 (d, *J* = 15 Hz, 2H).

#### Synthesis of compound 1

Reported literature is followed, for the sake of data completion, the procedure is presented here^17^.

4,4ʹ-bipyridine (4.0 g, 25.6 mmol) was dissolved in 20 ml CH_3_CN and stirred at 80 °C. To this solution 1-bromo ethane (0.56 g, 5.12 mmol) dissolved in (10 ml) CH_3_CN was added slowly over 8 h. The progress of the reaction was monitored by TLC (MeOH: HOAc: H_2_O –10:4:1). The reaction mixture was stirred for 24 h in total, and then cooled to room temperature. The reaction mixture was concentrated under reduced pressure and the resulting yellow precipitate was washed with excess diethyl ether to remove unreacted bipyridine. The bromide salt was then dissolved in minimal amount of water, precipitated with 3 M NH_4_PF_6_,filtered, washed and dried to yield the **1** as a white solid (1.42 g, 84%).^1^H NMR (400 MHz, CD_3_CN): δ, ppm 8.84 (dd, *J* = 6 Hz, 4H), 8.34 (d, *J* = 8.0 Hz, 2H), 7.82–7.81 (m, 2H), 4.63 (q, *J* = 6 Hz, 2H), 1.66 (t,*J* = 8 Hz, 3H). ^13^C NMR (100 MHz, CD_3_CN): δ, ppm 154.7, 151.9, 145.5, 142.1, 126.8, 122.6, 118.2, 57.8, 16.4. Anal. Calcd for C_12_H_13_F_6_N_2_P: C, 43.65; H, 3.97; N, 8.48. Found C, 43.55; H, 3.86; N, 8.41.

#### Synthesis of compound 2

*p*-xylylene dibromide (2.0 g, 7.57 mmol) was dissolved in 10 mL CH_3_CN and heated to 80 °C under stirring. To this solution, **1** (0.50 g, 1.51 mmol) dissolved in CH_3_CN (10 mL) was added slowly over 8 h. The reaction mixture was stirred for 48 h in total, and then cooled to room temperature. The reaction mixture was concentrated under reduced pressure and the resulting yellow precipitate was washed with excess diethyl ether to remove unreacted dibromide. The bromide salt was then dissolved in minimal amount of water, precipitated with 3 M NH_4_PF_6_, filtered, washed and dried to yield compound **2** as a white solid (0.75 g, 75%).(0.64 g, 64%). ^1^H NMR (400 MHz, CD_3_CN): δ, ppm 8.94 (dd, *J = *8 Hz, 4H), 8.37 (d, *J* = 8 Hz, 4H), 7.57–7.47 (m, 4H), 5.81 (s, 2H), 4.66 (q, *J* = 8 Hz, 2H), 4.61 (s, 2H), 1.64 (t, *J* = 8 Hz, 3H) ^13^C NMR (100 MHz, CD_3_CN) δ, ppm 151.3, 150.8, 146.4, 146.2, 146.1, 141.2, 133.5, 131.0, 130.5, 128.3, 128.0, 85.0, 58.5, 33.4, 16.4. Anal. Calcd for C_20_H_21_BrF_12_N_2_P_2_: C, 36.44; H, 3.21; N, 4.25. Found C, 36.42; H, 3.09; N, 4.00.

### Synthesis of compound EDV

Mixture of **1** (0.40 g, 1.50 mmol) and *p*-xylylene dibromide (0.18 g, 0.68 mmol) were dissolved in 25 ml CH_3_CN and stirred at 80 °C for 2 days. Then the reaction mixture was cooled to RT, and the precipitate was filtered and washed with CH_3_CN and dried. It was then dissolved in water, precipitated with NH_4_PF_6_, filtered, washed and dried to yield **EDV** (0.46 g, 64%). ^1^H NMR (400 MHz, CD_3_CN): δ, ppm 8.93 (dd, *J* = 4 Hz, 8H), 8.40–8.36 (m,8H), 7.59 (s, 4H), 5.85 (s, 4H), 4.67 (q, *J* = 8 Hz, 4H), 1.65 (t, *J* = 8 Hz, 3H). ^13^C NMR (100 MHz, CD_3_CN) δ, ppm 151.6, 150.7, 146.6, 135.3, 131.3, 128.6, 128.3, 128.1, 65.0, 58.7, 16.5. Anal. Calcd for C_32_H_38_F_24_N_4_O_2_P_4_.3H_2_O: C, 35.24; H, 3.51; N, 5.14. Found. C, 35.18; H, 3.49; N, 5.08.

### Synthesis of compound ETV

Mixture of 1,3,5-tris(bromomethyl)benzene (0.5 g, 1.40 mmol) and (2.0 g, 4.62 mmol) **1** were dissolved in 10 ml CH_3_CN and the reaction mixture was stirred at 80 °C for 24 h. Then the reaction mixture was cooled to RT, and the precipitate was filtered and washed with CH_3_CN and dried. It was then dissolved in water, precipitated with NH_4_PF_6_, filtered, washed and dried to yield the **ETV** (1.55 g, 72%). ^1^H NMR (400 MHz, CD_3_CN): *δ*_,_ ppm 8.96–8.91 (m, 12H), 8.43–8.37 (m, 12H), 7.67–7.52 (m, 3H), 6.32 (s, 6H), 5.83 (s, 6H), 4.67 (q, *J* = 16 Hz, 6H), 1.64 (t, J = 12 Hz, 9H). ^13^C NMR (100 MHz, CD_3_CN) δ, ppm 151.8, 150.3, 146.7, 146.3, 132.8, 132.1, 128.4, 128.3, 128.1, 128.1, 64.5, 58.6, 16.5. Anal. Calcd for C_45_H_48_F_36_N_6_P_6._: C, 35.03; H, 3.14; N, 5.45. Found C, 34.89; H, 3.06; N, 5.33.

### Synthesis of compound ESV

Mixture of **2** (0.35 g, 0.52 mmol) and **P0** 3PF_6_^34^ (0.15 g, 0.15 mmol) were dissolved in 20 ml CH_3_CN, stirred at 80 °C for 4 days. The progress of the reaction was monitored by TLC. Then the reaction mixture was cooled to RT, filtered, washed with CH_3_CN and dried. It was then dissolved in water: MeOH (1:1) mixture, precipitated with 3 M NH_4_PF_6_, filtered, washed and dried to yield **ESV**(0.31 g, 65%). ^1^H NMR (400 MHz, CD_3_CN): δ, ppm 8.99–8.94 (m, 24H), 8.43–8.42 (m, 24H), 7.67 (s, 2H), 7.62 (s, 13H), 5.88–5.85 (m, 18H), 4.70 (q, *J* = 6 Hz, 6H), 1.68 (t, *J* = 8 Hz, 9H). 13 C NMR (100 MHz, CD_3_CN): δ, ppm 151.5, 151.4, 151.3, 150.6, 146.6, 146.5, 146.2, 146.1, 135.7, 135.2, 135.1, 132.6, 131.2, 128.3, 128.0, 127.3, 123.4, 64.8, 64.4, 58.5, 16.4. Anal. Calcd for C_99_H_96_F_72_N_12_P_12_.2H_2_O: C, 36.82; H, 3.12; N, 5.20. Found C, 36.75; H, 2.96; N, 5.09.

### UV-Vis

In UV-Vis, dialkyl bipyridinium salts (viologens) typically show an absorption maxima at 250 nm approx. corresponding to the dication, and its one electron reduced monocation radical (V^+•^) shows an absorption maxima at 400 and 600 nm respectively^37^. This monocation radical has the tendency to dimerize i.e., formation of intramolecular charge transfer complex between the reduced monocation radicals and there is an equilibrium between the monocation radical and its dimeric form. A typical UV-Vis measurement shows absorption maxima at 400, 600 nm corresponding to the monocation radical, upon complexation the wavelength shifts to 550 nm (blue shift)^38^ corresponding to the monocation radical and an additional 800–1100 nm corresponding to the dimer is observed^16,37,39^. The ratio between these two bands indicates the degree of dimerization.

**Figure 3:**
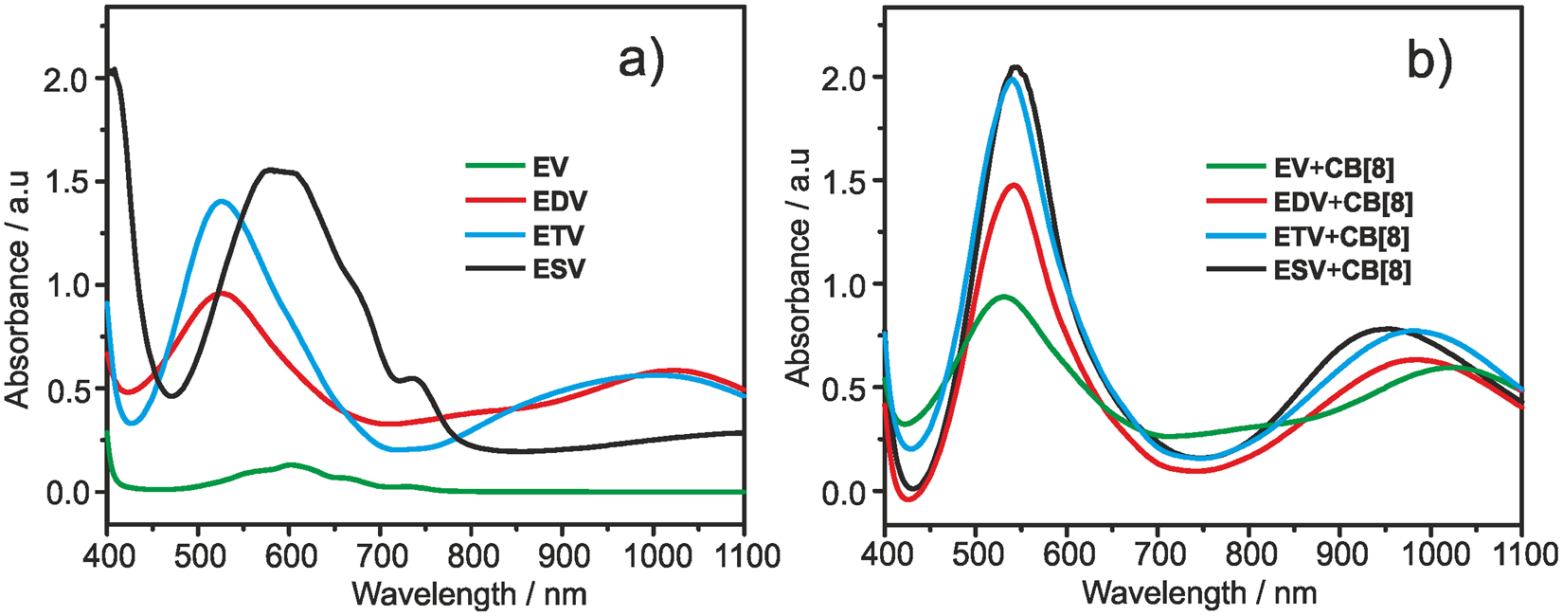
The absorption spectrum of (**a**) **EV**, **EDV**, **ETV** & **ESV** (0.25 mM) and (**b**) the absorption spectrum in the presence of CB[8], [CB[8]] = 1.0, 2.0, 3.0 and 6.0 equiv. for **EV**, **EDV**, **ETV** & **ESV** respectively) in sodium phosphate buffer (0.1 M) solution containing sodium dithionite (50 mM), RT.

Ethyl viologen (**EV**) is used as a model compound in this work. UV-vis spectra of four different ethyl terminated viologen compounds (**EV**, **EDV**, **ETV & ESV**) were recorded in aqueous phosphate buffer (pH = 7) solution containing excess sodium dithionite (50 mM) and are displayed in Fig. 3. [In aqueous solution, sodium dithionite readily reduces viologen (V^2+^) to monocation radical (V^+•^)]. All the viologen radical cations (**EDV**^**2+•**^, **ETV**^**3(+•)**^, **ESV**^**6(+•)**^ except **EV**^**(+•)**^) displayed broad and strong absorption bands. **EDV**^**2(+•)**^ showed absorption maxima at 525 and 1020 nm, **ETV**^**3(+•)**^ at 525 and 990 nm, **ESV**^**6(+•)**^ at 590 and >1100 nm corresponding to the monocation radical and its dimer respectively. In the case of **EV**^**(+•)**^, an absorption maxima centered around 600 nm corresponding to the monocation radical was observed (Fig. 3a) and this indicates that the monomer behaves differently than that of the branched architectures. In the case of branched architectures, because of the flexible benzyl interconnection, dimerization is more favored and hence intra/intermolecular CT complexation occurs. All these results strongly suggest that except the monomer **EV**^**(+•)**^, all the other branched viologens (**EDV**^**2(+•)**^, **ETV**^**3(+•)**^, **ESV**^**6(+•)**^) undergo dimerization. In the presence of CB[8], the affinity to form radical dimer (V^+•^)_2_ increases as the dimer can be easily encapsulated into the hydrophobic cavity of CB[8] which is additionally stabilized by ion-dipole interactions between the oxygen atoms (16 nos) and radical cation^26,40^. On the other hand, the possibility for intramolecular stacking between radical cations of peripheral viologen units in the projected viologen derivatives is fewer as the intramolecular radical dimerization is mainly distant dependent^16^. Further the presence of rigid benzene core moiety and unfavorable steric interactions suppress the possibilities of intramolecular dimerization.

Figure 3b shows the UV-Vis spectra of CB[8] encapsulated viologen radicals. It is clear that all the four molecules are included in the CB[8] cavity in their dimerized form as the intensity of the absorption band at 800–1100 nm is more strong than their corresponding monocation radical spectrum without CB[8]. Even the monocation radical of **EV** shows an intense band at 1020 nm indicating that it is included in the CB[8] cavity in the dimerized form. These results clearly suggest that in the presence of CB[8] dimerization is more favored and such dimerization can be a result of inter/intramolecular interactions. It is important to note that intramolecular dimerization inhibit the formation of supramolecular polymer. However, the particle size measurements show that we have larger aggregates indicating that they are formed from a long chain linear/hyperbranched supramolecules. Such supramolecular polymerization is a result of intermolecular dimerization between the molecules.

The association constants of all the four molecules were estimated from UV-Vis titrations and the plots are given in the supporting information. Table 1 depicts the physical chemical data of all the four viologen derivatives.

**Table 1:** Physico-chemical data of ethyl terminated viologen radical cations (EV^(+•)^, EDV^2(+•)^, ETV^3(+•)^ and ESV^6(+•)^).

**V** ^**(+•)**^	**Cyclic voltammetry**					**Association constant**, ***K***_**a**_ **(presence of CB[8])**, **M**^**−1**^	**Particle size (before and after addition of CB[8]**, **nm**	
	**Absence of CB[8]** E_pc1/_ E_pa1_^I^E_pc2/_ E_pa2_^II^ **(V)**		**Presence of CB[8]** E_pc1/_ E_pa1_ E_pc2/_ E_pa2_ E_pc3/_E_pa3_^III^ **(V)**				**Before**	**After**
**EV** ^**(+•)**^	−0.62/−0.55	−0.89/−0.76	−0.47/−0.37	−0.62/−0.55	−0.90/−0.81	8.9 × 10^3^	661	1356
**EDV** ^**2(+•)**^	−0.38/−0.33	−0.71/−0.58	−0.41/−0.26	−1.10/−0.68	—	4.7 × 10^3^	535	720
**ETV** ^**3(+•)**^	−0.38/−0.32	−0.73/−0.61	−0.27/−0.19–0.40/−0.33	−0.79/−0.66	−0.40/−0.33	6.2 × 10^3^	857	1045
**ESV** ^**6(+•)**^	−0.31/ −0.22	−0.76/−0.52	−0.41/−0.23	−0.76/−0.59	—	2.1 × 10^4^	351	1362

^I,II,III^First, second and third redox potentials of viologen radical cations.

### EPR

**Figure 4:**
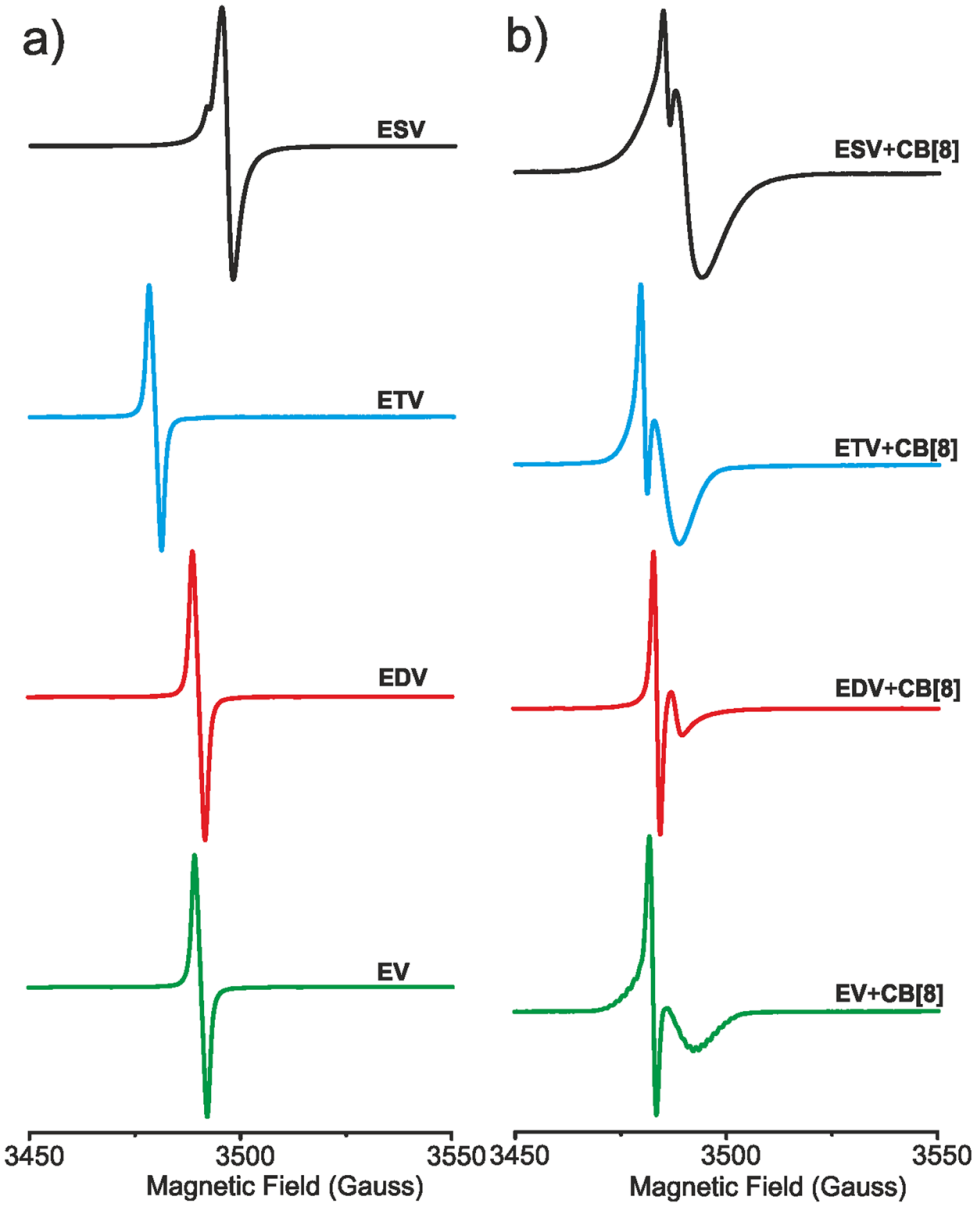
EPR spectra of **EV**, **EDV**, **ETV** & **ESV** (0.50 mM) in sodium phosphate buffer (0.1 M) containing sodium dithionite (50 mM) (**a**) in the absence and (**b**) in the presence of CB[8], RT. (CB[8] = 1.0, 2.0, 3.0 and 3.0 equiv. for **EV**, **EDV**, **ETV** and **ESV** respectively).

Electron paramagnetic resonance spectroscopy (EPR) gives clear insight on the material with unpaired electrons. Since we reduced the viologen dication to monocation radical and encapsulated them into the CB[8] cavity, EPR analysis can probe the nature of the radicals and their interaction with CB[8]. In this work, we have investigated the paramagnetic properties as well as the dimerization tendency of four different viologen radical cations such as **EV**^**(+•)**^, **EDV**^**2(+•)**^, **ETV**^**3(+•)**^ & **ESV**^**6(+•)**^. The concentration of the monocation radical (0.50 mM) and CB[8] (0.25 mM) were kept constant throughout the experiment. EPR spectra were recorded in sodium phosphate buffer solution (0.1 M) containing sodium dithionite (50 mM). The radical cation of the model compound (EV^(+•)^) showed a sharp EPR signal with a *g-*factor value 2.0046 (Fig. 4a) closer to that of a *g*-factor of free electron (*g* = 2.0023)^31^ and this is in agreement with the reported values for simple viologen monocation radical indicating the very organic feature of the radical^27,28,32^. The sharp hyperfine splitting of respective viologen radical cations (**EV**^**(+•)**^, **EDV**^**2(+•)**^, **ETV**^**3(+•)**^ **& ESV**^**6(+•)**^) indicated that the radical cations (here after denoted as (V^+•^)) exclusively gets dimerized (V^+•^)_2_ as reported by Zhang *et al*.^27^. However, all these molecules **EV**^**(+•)**^, **EDV**^**2(+•)**^, **ETV**^**3(+•)**^ **& ESV**^**6(+•)**^ displayed a different behavior than the molecules reported by Zhang *et al*. EPR spectra of all these radicals displayed hyperfine splitting with high resolution corresponding to a dimer, and we could not detect any weak broad hyperfine signal corresponding to the monocation radical very closer to the radical dimer except in the case of **ESV**^**6(+•)**^ (a small hump is seen) before the addition of CB[8]^27^. These results clearly indicate that the monocation radicals exclusive dimerize under our experimental conditions. Nevertheless, in the case of EV, these results are in conflict with the UV-Vis measurements and this could be related to the equilibration time. Probably the EV^(+•)^ needs more time for the complex formation and equilibration time under UV-Vis measurement conditions is insufficient.

Under identical conditions CB[8] was added to the solution containing radical dimer, equilibrated and EPR spectrum was recorded as displayed in Fig. 4b. The presence of CB[8] developed a weaker broad hyperfine splitting for the dimer (V^+•^)_2_. These broad hyperfine structures specify the encapsulation of viologen dimeric species into the hydrophobic cavity of CB[8]. As a result of encapsulation, electron-electron exchange interaction between the corresponding viologen radicals becomes reduced (dipole-dipole interactions becomes weak)^38^. The strength of the broad hyperfine structure increases in the order **EDV**^**2(+•)**^ > **EV**^**(+•)**^ > **ETV**^**3(+•)**^ > **ESV**^**6(+•)**^ (Fig. 4b) indicating the decrease in concentration of dimer species (V^+•^)_2_ upon complexation with CB[8]. All these results clearly indicate the existence of intermolecular- interactions between the radical dimers (V^+•^)_2_ which is further enhanced in the presence of CB[8] as indicated by UV-Vis and EPR measurements. Intermolecular interactions in these structure leads to molecular self-assembly in a 2D (**EDV**^**2(+•)**^, **ETV**^**3(+•)**^, **ESV**^**6(+•)**^) orientation. Addition of CB[8] further enhances the formation of inclusion complexes leading to the construction of a well-defined 2D supramolecular polymers as reported by Zhang *et al*.^27^ and Marchini *et al*.^32^. These self-assembled architectures are guided through non-covalent interactions and their consequent movements and rotations indicate that the supramolecular network should be polydisperse and hence the order of these polydisperse supramolecular polymers may be ideal or randomly arranged 2D structures^26^.

### Cyclic voltammetry

Viologens or 4,4′-bipyridinium salts exhibit excellent redox behavior and undergoes two consecutive one electron reduction to form the monocation radical and neutral bipyridinylidene. A standard dialkyl viologen shows redox peaks at −0.4 V and −0.8 V corresponding to the reduction of dication to monocation radical (V^2+^ ↔ V^+•^) and monocation radical to neutral bipyridinylidene (V^+•^ ↔ V^0^) respectively. The electrochemical potential required for the redox process of viologens solely depends on N-substitution^16^.

**Figure 5:**
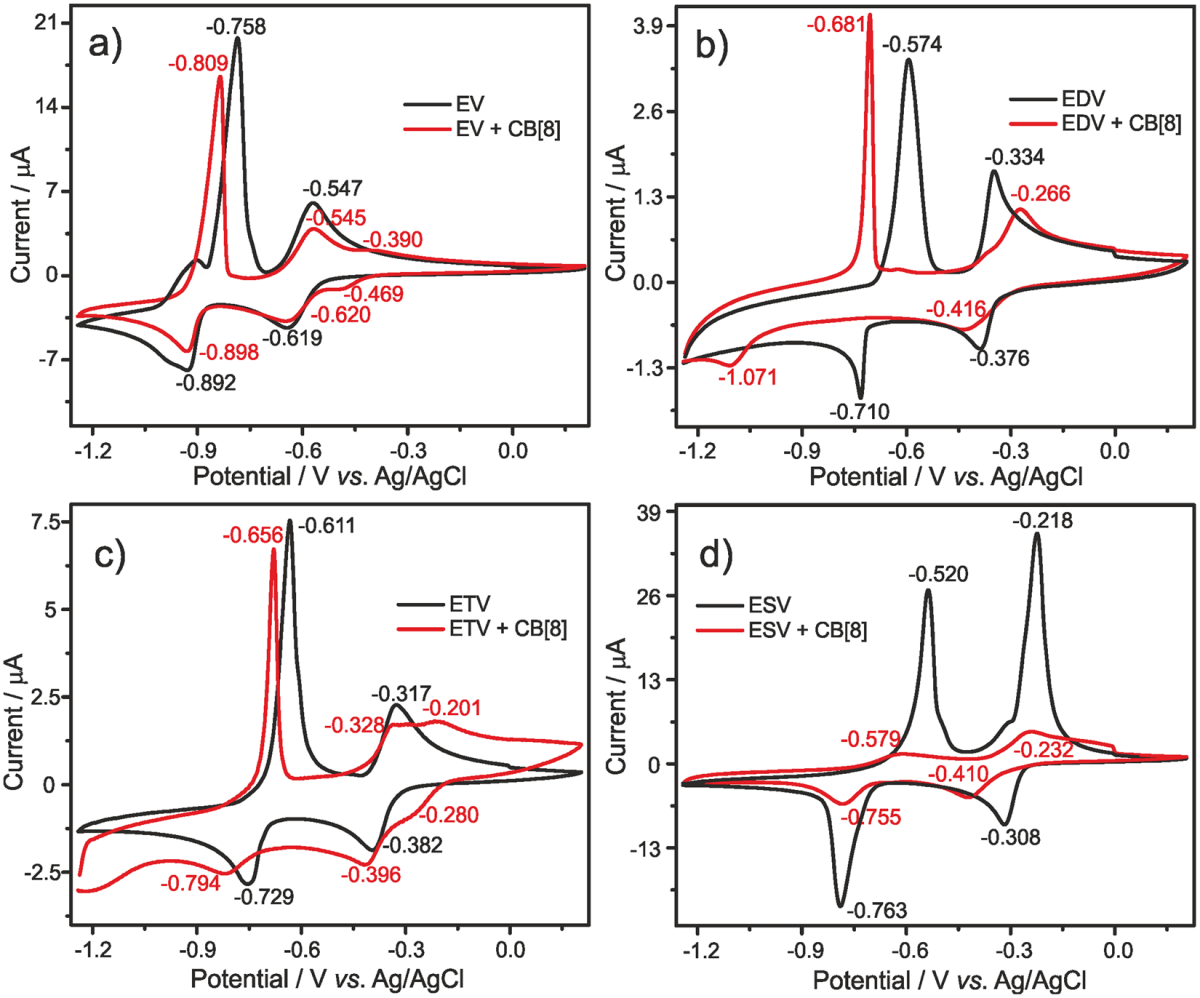
Cyclic voltammograms of Viologen derivatives (**a**) **EV**, (**b**) **EDV**, (**c**) **ETV** and (**d**) **ESV** (1.0 mM) in aqueous KCl (0.1 M) on glassy carbon (0.07 cm^2^) in the absence (black line) and the presence (red line) of CB[8] (1.0 mM, (1.0 equiv. for all viologens)) at *V* = 0.1 V/s, RT.

The electrochemical properties of four different ethyl terminated viologens **(EV**, **EDV**, **ETV** & **ESV)** and their inclusion complexation with CB[8] were monitored by cyclic voltammetry (CV) (Fig. 5a–d) in the presence and absence of CB[8]. Model compound ethyl viologen **(EV)** displayed two reduction peaks at a potential of −0.62 V and −0.89 V. Further the positive and negative potential shifts for the rest of the three viologen compounds were compared with redox potential values of **EV**. Typically the first reduction, V^2+^ ↔ V^+•^ occurs easily, while the second reduction (V^+•^ ↔ V^0^) becomes more difficult, as more electrical stimuli is required for reducing the monocation radical (V^+•^) [there is an equilibrium between the monocation radical and its dimerized form (V^+•^ ↔ V^+•^- V^+•^)]. Thus the difficulty in the second reduction i.e., larger shift in the electrochemical potential can be attributed to the dimerization of the monocation radical. The electrochemical potential difference between the two reduction and two oxidation waves in ethyl terminated viologens were investigated by CV, and the potential differences were denoted as ∆E_p_, ∆E_p_ increases with increase in 4,4′-bipyridinium units in the respective viologen architectures **(EV**, **EDV**, **ETV** and **ESV)**.

Notably, the reduction potential differences were 0.27 V for **EV**, 0.33 V for **EDV**, 0.35 V for **ETV** and 0.46 V for **ESV** and these shift in potential differences enhance the stability of their respective dimer (V^+•^)_2_. On the other hand, the anodic potential increased in the order **EV** (0.21 V) < **EDV** (0.24 V) < **ETV** (0.29 V) < **ESV** (0.30 V) and such increments can be accounted for the additional stabilization of the dimer. It is noteworthy that in the absence of CB[8], all the four viologens displayed similar CV profile and diffusion behavior. In the presence of CB[8], viologens compounds displayed different behavior, **EDV** and **ESV** showed same number of redox waves with obvious positive and negative redox potential shifts. Particularly **EDV** showed larger ∆E_p_ values of 0.42 V and 0.66 V for the corresponding anodic and cathodic potential differences in the presence of CB[8]. These large difference in the reduction potential could be ascribed to the higher tendency of radical dimerization (V^+•^)_2_ in the presence of CB[8] and further stacking of such inclusion assembly as probed by particle size measurements. Noteworthy is the very low diffusion current of the **ESV-CB[8]** complex (approx. 5–7 times lesser) compared to that of the parent compound (**ESV**) depicting the fact that it forms large molecular aggregates which limits its self-diffusion towards the electrode surface. In all the other cases decrease in diffusion current of approx. 1.5–2 times was observed indicating the formation of inclusion complex. With and without CB[8], the first oxidation peak in all the four cases depicts preferential adsorption of the complex over the electrode surface (except **ESV-CB[8]**complex which shows normal redox pattern). The other viologens such as the model compound **EV** and the trimer **ETV** showed three different reduction waves, wherein the first two reduction peaks are ascribed to the **EV**^**2+**^** ↔ EV**^**+•**^ and **EV**^**2+**^** + EV**^**+•**^ → **(EV**^**+•**^**)**_**2**_ processes^27^. The third reduction peak is attributed to the conversion of radical cation to its neutral form **EV**^**+•**^** ↔ EV**^**o**^ at the potentials of −0.89 (**EV)** and −0.79 (**ETV**). The slight potential shifts on both anodic as well as cathodic side is a result of an improved stability of (V^+•^)_2_ after encapsulation into hydrophobic cavity of CB[8].

### Particle size measurements

**Figure 6:**
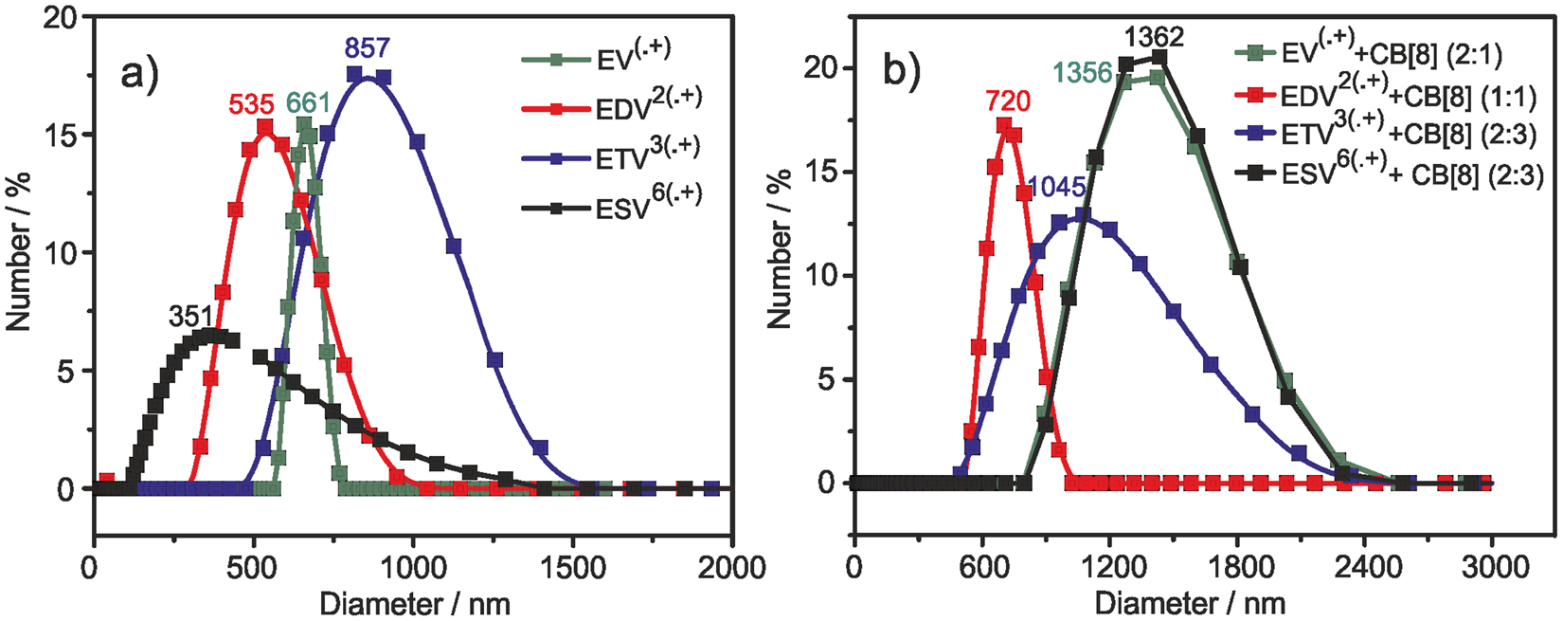
Particle size analysis of **EV**, **EDV**, **ETV** & **ESV** (0.2 mM), measured in aqueous solution containing excess of sodium dithionite (**a**) in the absence and (**b**) in the presence of CB[8] (0.1 mM), RT.

UV-Vis, EPR and cyclic voltammetric studies clearly revealed the possibility of formation of intermolecular dimerized supramolecular network where the arrangement on 2D depends on the nature of the viologen precursor **(EV**, **EDV**, **ETV** & **ESV)** with CB[8]. To infer more details into the supramolecular framework, particle size measurements were done for viologen compounds without and with CB[8] in aqueous solutions. These supramolecular framework were constructed by self-stacking the respective viologen radical cations (V^+•^) and they exhibit certain hydrodynamic diameter (*D*_H_) (particle size) in water. The *D*_H_ value of supramolecular entity formed by (V^+•^) before the addition of CB[8] was found to be 0.66 μm for **EV**, 0.53 μm for **EDV**, 0.85 μm for **ETV** and 0.35 μm for **ESV** (Fig. 6). These results clearly specify that the compounds are stacked in to very larger aggregates guided by molecular self-assembly and these results are in contrast to the recent reports on supramolecular assemblies where particle size of several nm were reported^27,28^. The larger aggregates/polydispersely arranged network could stack to form 2D supramolecular assemblies as reported by Zhou *et al*., Zhang *et al*. and Marchini *et al*.^27,28,32^. In the presence of 0.1 mM CB[8], the aqueous solution containing excess of sodium dithionite and **EV**^**(•+)**^ produced aggregates with the *D*_H_ value 1.35 μm which was twice when compared with the value (0.66 μm) without CB[8], denoting that 2:2 or 1:1 complex was formed in the mixture. Further the solution containing 0.2 mM CB[8] and **EDV**^**2(•+)**^ formed aggregates with the *D*_H_ value of 0.72 μm which is slightly larger compared to the value (*D*_H_ = 0.53 μm) without CB[8]. This smaller difference in particle size indicates the formation of 2:1 complex between CB[8] and **EDV**^**2(•+)**^ in water. On the other hand in the presence of 0.3 mM CB[8], the solution containing dimerized species of **ETV**^**3(•+)**^, **ESV**^**6(•+)**^ produced aggregates with the *D*_H_ values of 1.04 μm and 1.03 μm respectively. These *D*_H_ values of un-complexed **ETV**^**3(•+)**^ and **ESV**^**6(•+)**^ species were 0.85 μm and 0.35 μm. These particle size differences indicates the formation of 2:1 and 2:3 complexes of **ETV**^**3(•+)**^ and **ESV**^**6(•+)**^ with CB[8] respectively.

In addition, zeta potential measurements were carried out to study on the stability of these monocation radicals in the absence and presence of CB[8] (Figure S1). The stability of a colloidal dispersion can be described by zeta potential. In the absence of CB[8], we obtained zeta potential values of −5.8, −3.0, −10.7 and −18.6 mV and in the presence of CB[8], 5.8, 2.4, 9.3 and 15.8 mV were obtained for **EV**^**(•+)**^, **EDV**^**2(•+)**^, **ETV**^**3(•+)**^ and **ESV**^**6(•+)**^ respectively. All these values indicate high instability/rapid aggregation of these molecules. It is noteworthy that in the absence of CB[8], the zeta potential values were negative and as soon as the intermolecular dimer gets encapsulated in CB[8] cavity, the value becomes positive.

### TEM & HR-TEM analysis

**Figure 7:**
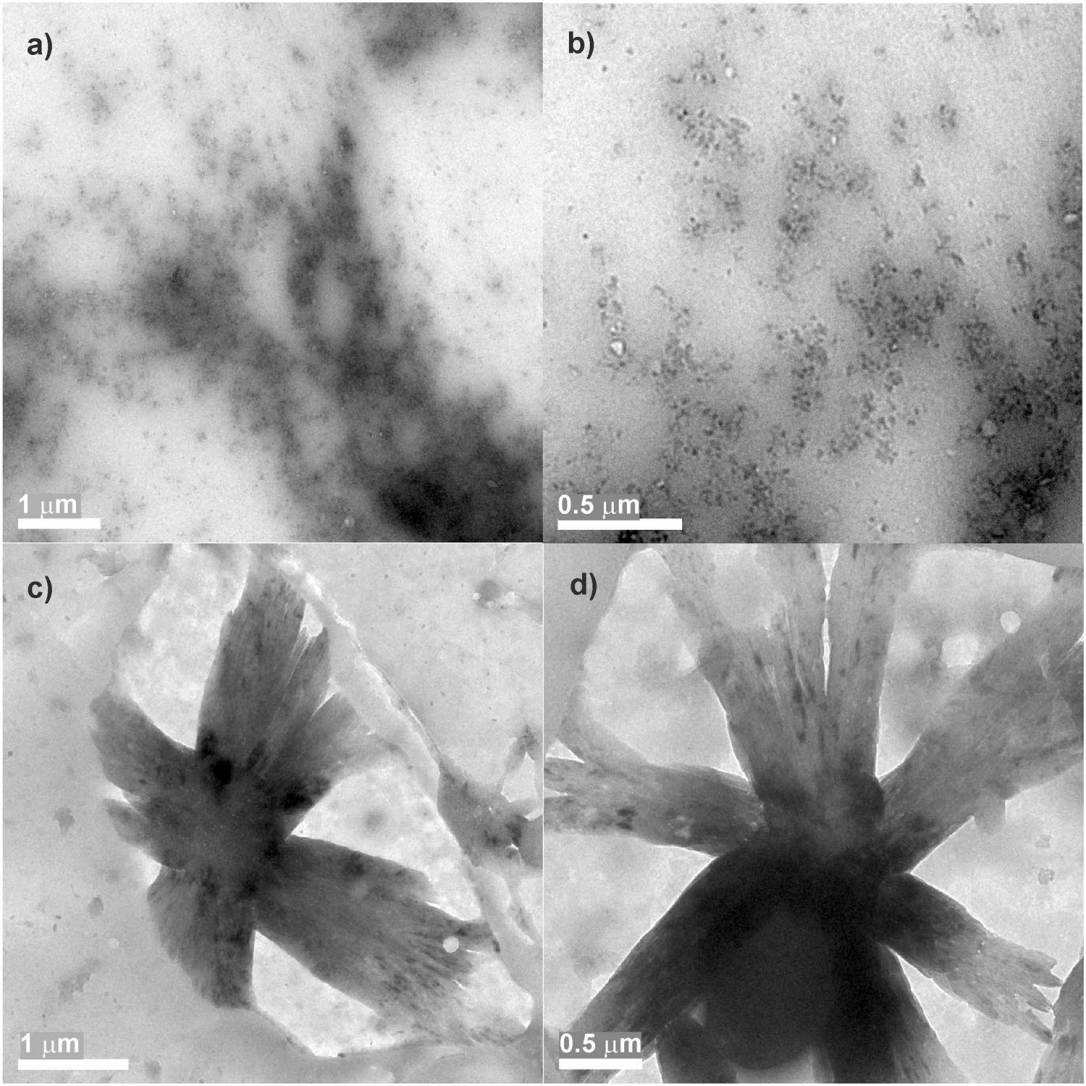
TEM images of **ETV** (0.1 mM) + **CB[8]** (3 equiv.) before (**a**,**b**) and after reduction (**c**,**d**) with sodium dithionite.

TEM (transmission electron microscope) analysis of viologen trimer further revealed the formation of supramolecular polymer (**ETV**^**3(•+)**^ with cucurbit[8]uril) whose diameter were in the range of several µm as indicated by DLS measurements; however the oxidized form, i.e., the viologen dication with cucurbit[8]uril showed dotted spots in the range of sub nanometer level (Fig. 7). It is very clear from TEM experiments that these samples show a characteristic difference in their morphology before and after reduction.

HR-TEM images of the samples were given in supporting information (Figures S10 and S11). SAD (selected area diffraction) analysis by HR-TEM reveals the semi-crystalline nature of these supramolecular polymers (**EDV**, **ETV** and **ESV**).

### SAXs measurements

**Figure 8:**
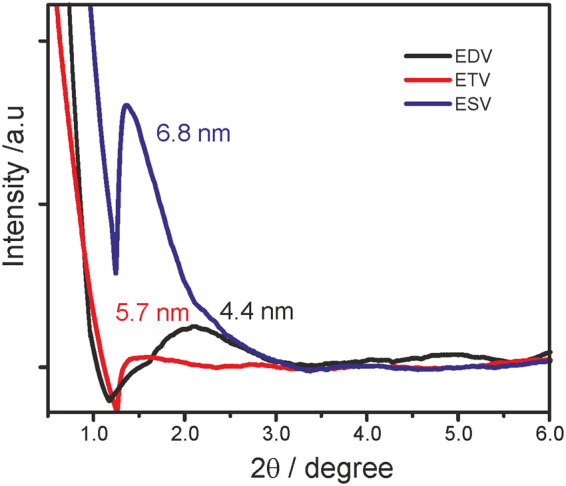
Thin layer SAXs profile of **EDV**, **ETV**, **ESV** (*c* = 0.2 mM) in the presence of CB[8] (0.1 mM) and sodium dithionite.

The internal periodicities of the samples were measured by SAXs (small angle X-ray scattering) measurements and are shown in Fig. 8. The following conclusions can be drawn from the SAXs measurements

(i) **EDV** showed a broad scattering peak with a d-spacing value of 4.4 nm corresponding to {200} facet^41^.
(ii) **ETV** showed relatively less scattered broad peak with a d-spacing value of 5.7 nm corresponding to {100} facet
(iii) **ESV** showed a sharp peak with a d-spacing value of 6.8 nm corresponding to {100} facet^41^.

The internal periodicities of these samples clearly reflect the fact that they form 2D architecture^27^. This is further confirmed by SAD pattern measured from the TEM analysis.

**Figure 9:**
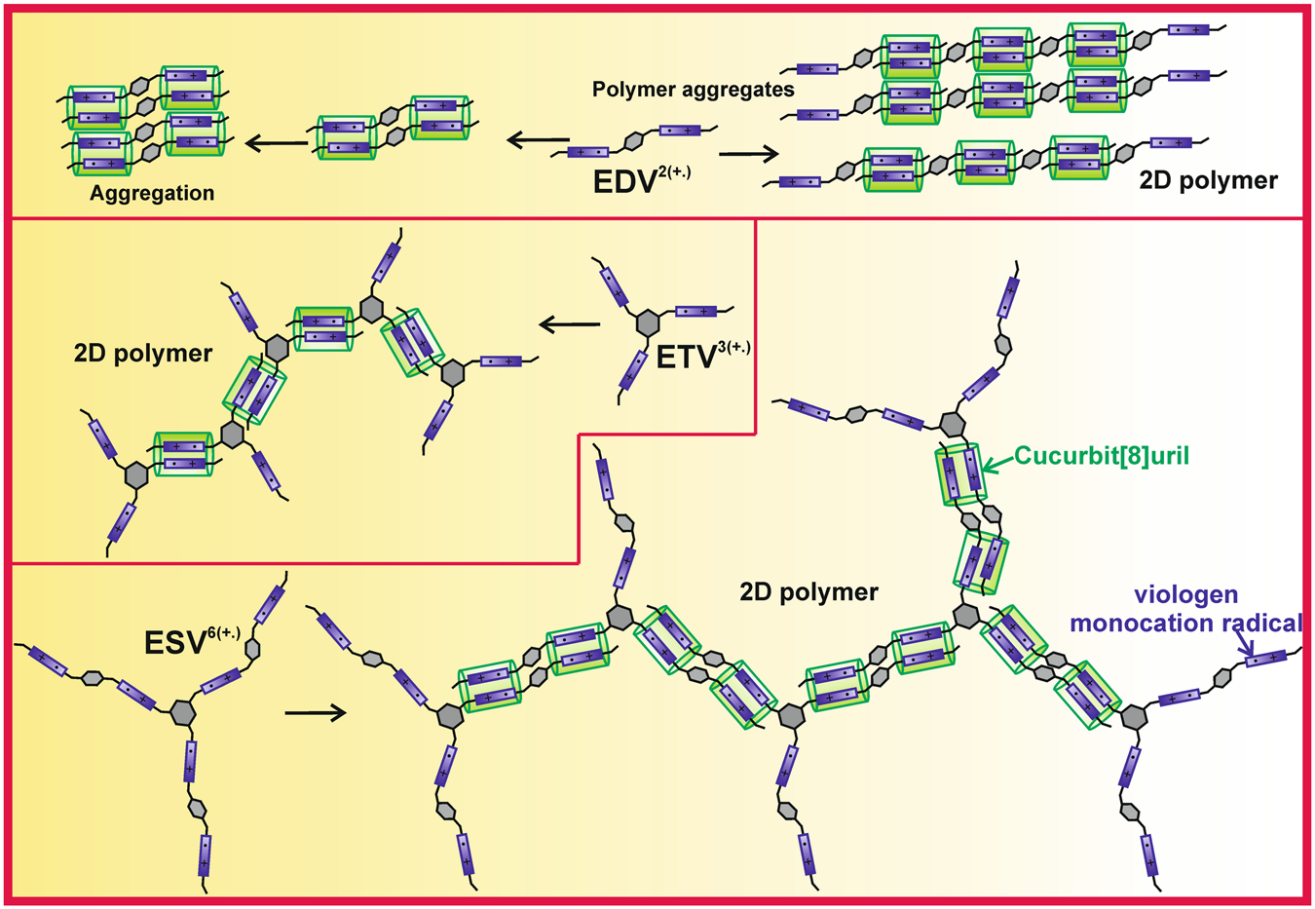
Scheme depicting the supramolecular self assembly process involved in **EDV**, **ETV** and **ESV**.

Based on UV-Vis studies, SAXs measurements and particle size measurements, we propose the following scheme to illustrate the supramolecular self-assembly process as shown in Fig. 9. The whole process is accompanied by two key processes, i) supramolecular polymer formation and ii) aggregation. The model compound diethyl viologen is involved in dimer formation and aggregation, whereas all the other three compounds form supramolecular polymer network and its aggregates. For simplicity, only for dimer we have given the mechanism of aggregate formation and whole mechanism, in the case of trimer and star shaped viologens, the mechanism is complex and the pictographical representation of such complex would be tedious.

### Reversibility Studies by UV-Vis spectroscopy

It is well-known that the viologen dication undergoes two electron reductions to produce monocation radical and a neutral species and such reduced species can be reoxidized chemically/electrochemically. Throughout this study, we employed reduced monocation radical to study its dimerization and subsequent formation of supramolecular organic framework. Hence it is possible to reoxidize this species by aerial oxidation or by passing O_2_ gas.

**Figure 10:**
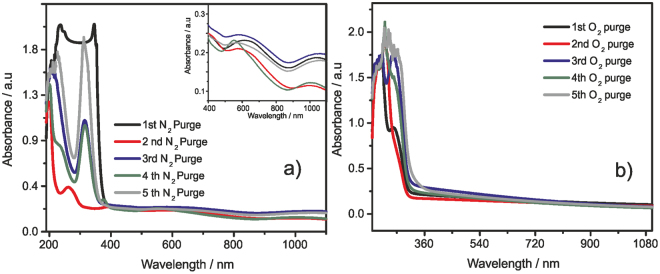
UV-vis reversibility study of **EDV** (0.25 mM) with CB[8] (2.0 equiv.) was monitored in the aqueous solution containing excess of sodium dithionite; (**a**) after degassing with N_2_; inset shows the absorption of dimer, and (**b**) after O_2_ bubbling, RT.

To probe more on the reversibility of supramolecular polymerization, we performed five subsequent cycles of UV-V is measurements by consecutive oxidation and reduction of the same solution (Fig. 10). The fully reduced solution containing viologen monocation radical/dimer V^+•^/V^(+•)^_2_ with CB[8] was oxidized by bubbling O_2_ and the spectra was recorded which showed typical viologen dication (V^2+^) absorption at 220–260 nm. This solution was again degassed with N_2_ and reduced with sodium dithionite. The corresponding UV-Vis spectra showed typical bands for the monocation radical and the dimerized species. Likewise, the experiment was repeated for five consecutive cycles and the system was found to be reversible. Thus, the supramolecular polymerization can be reversed by bubbling oxygen and can be formed again by degassing with N_2_ and reducing with sodium dithionite. Under our experimental conditions, we were able to repeat this cycle for at least five times without much decrease in the redox activity and hence we believe that these supramolecular polymerization can be reversed i.e., depolymerized. Once oxidized, the dicationic viologens exhibit electrostatic repulsion and hence they unwind from CB[8] cavity, where only one viologen unit remain encapsulated in the CB[8] cavity. Thus the system becomes monomeric. Again the reduction with sodium dithionite under inert atmosphere yields monocation radical which can undergo facile intermolecular dimerization into the CB[8] cavity forming supramolecular polymeric network. Thus oxidation leads to depolymerization of the network and subsequent reduction yields the supramolecular polymeric network.

## Conclusion

In summary, we reported the 2D supramolecular organic framework formed between the different viologen guest molecules **(EV**, **EDV**, **ETV** and **ESV)** and CB[8] and the interactions were probed spectroscopically in detail by employing UV-Vis spectroscopy, cyclic voltammetry, EPR spectroscopy and particle size measurements. The mechanism of self-assembly was: (i) strong stacking of viologen radical cations (V^+•^) in aqueous/phosphate buffer solution containing excess of sodium dithionite, (ii) the dimerized species (V^+•^)_2_ can be included in to hydrophobic cavity of CB[8] which additionally stabilizes the (V^+•^)_2_ species. UV-vis investigation clearly proves that the radical cations were involved in intermolecular dimerization and the dimerized species were encapsulated into CB[8] cavity. This inclusion behavior was nicely supplemented by UV-Vis spectroscopy in the region of 800–1100 nm. EPR studies also revealed that the addition of CB[8] enhanced the dimerization. Particle size measurements showed a particle size in the range of several µm indicating larger aggregates and strongly supported the formation of 2D single -assembly as reported by Zhou *et al*. and Zhang *et al*. However, our results partly vary from their previous reports in terms of particle size. Zeta potential measurements suggested that these particles are highly unstable and have the tendency to form aggregates readily, and notably zeta potential value changed from negative to positive upon encapsulation with CB[8]. In addition, we have demonstrated that these supramolecular organic frameworks can be depolymerized by oxidation in air and again can be polymerized (intermolecular radical dimerization) by reduction under inert atmosphere. Such reversible systems are of broad interest as they show advantages over irreversible systems. A key point of these newly–assembled processes are high stability of radical cations with formation of larger aggregates leading to the formation of 2D assembly as indicated by TEM. SAXs profile of the supramolecular polymer samples showed broad scattering peaks indicating the internal periodicity of the supramolecular polymer. Thus the dimerization of radical cations can be considered as a new strong non-covalent force to construct highly ordered supramolecular ensembles for wide range of applications. Future studies will focus on the photophysical and spectroelectrochemical investigations on these systems towards reversible electron/charge storage properties.

## Electronic supplementary material

Supplementary Information
