# Supplementary material for: Reversible 2D Supramolecular Organic Frameworks encompassing Viologen Cation Radicals and CB[8]
Source: Sci Rep. 2018 Jan 22;8:1354. doi: 10.1038/s41598-018-19739-7 (PMC5778010; doi:10.1038/s41598-018-19739-7)
Supplement: Supplementary file 1 — Supplementary Information [file 41598_2018_19739_MOESM1_ESM.pdf]

Supporting information

Reversible 2D Supramolecular Organic Frameworks encompassing Viologen Cation Radicals and CB[8]

Kanagaraj Madasamy,[a, b] Vellaiah Maruthiah Shanmugam,[a] David Velayutham,[a,b] Murugavel Kathiresan*[a,b]

[a)] Electroorganic Division, CSIR-Central Electrochemical Research Institute, Karaikudi-630003, TamilNadu, INDIA. E-mail: kathiresan@cecri.res.in

[b)]Academy of Scientific and Innovative Research (AcSIR), CSIR-Central Electrochemical Research Institute, Karaikudi-630003, TamilNadu, INDIA

Binding constant

Binding constants were estimated using Benesi–Hildebrand method following a reported procedure. [CB[8]] = 1.0, 2.0, 3.0 and 6.0 equiv. for **EV**, **EDV**, **ETV** & **ESV** respectively.

Ethyl viologen (model compound, EV)

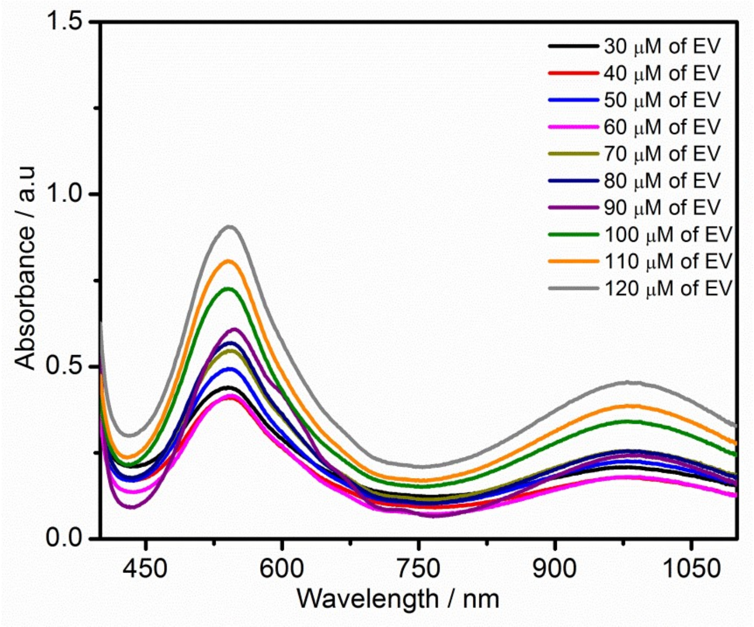

Fig S1. Absorption spectrum of **EV$^{(+\bullet)}$ - CB[8].**

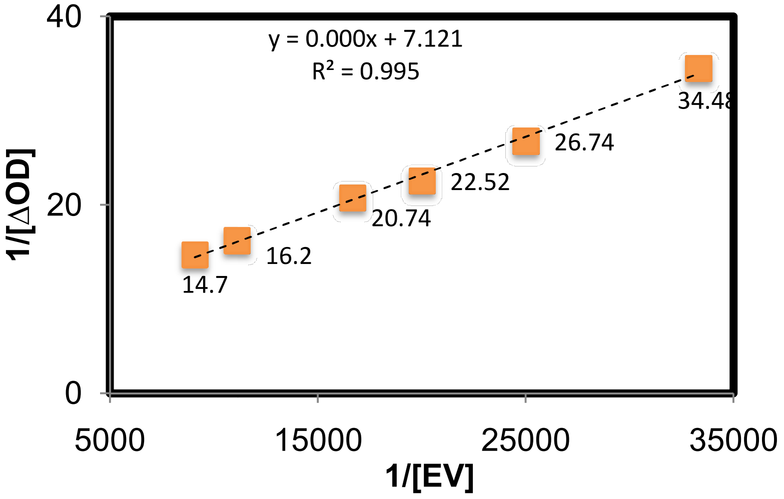

Fig S2. Benesi-Hildebrand plot of **EV$^{(+\bullet)}$- CB[8]** titration by UV-visible spectrum.

Binding constant (K_a) value for complexed **EV** was 8.9 X 10^3 M^{-1}.

Viologen Dimer (EDV)

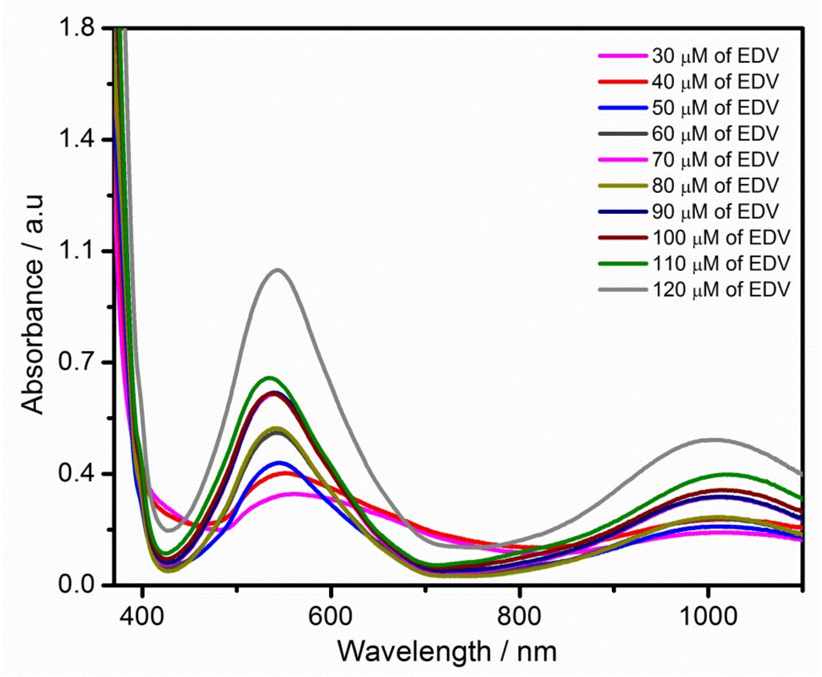

Fig S3. Absorption spectrum of **$EDV^{2(+\bullet)}$ - CB[8].**

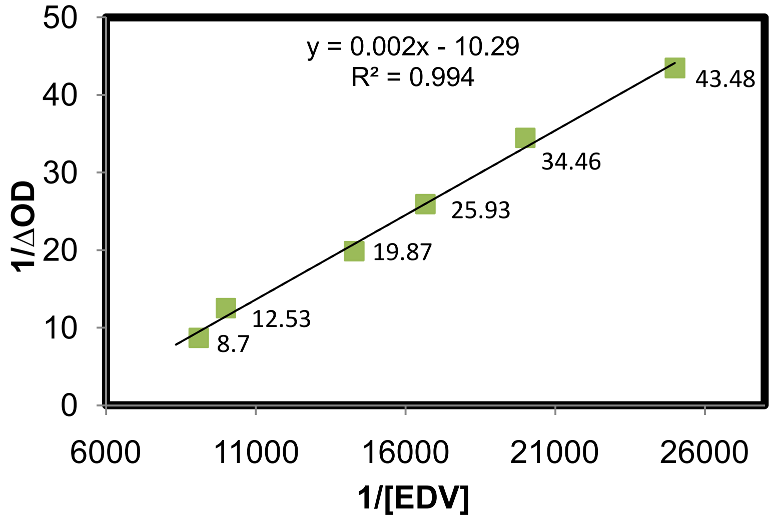

Fig S4. Benesi-Hildebrand plot of **$EDV^{2(+\bullet)}$ - CB[8]** titration by UV-visible spectrum.

Binding constant (K_a) value for complexed **EDV** was 4.7 X 10^3 M^{-1}

Viologen Trimer (ETV)

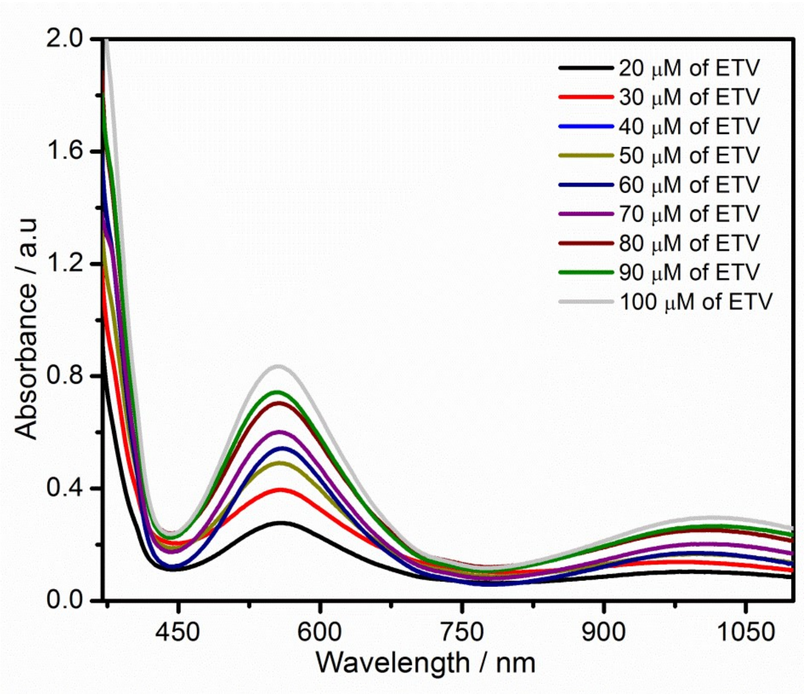

Fig S5. Absorption spectrum of **ETV$^{3(+\bullet)}$ - CB[8].**

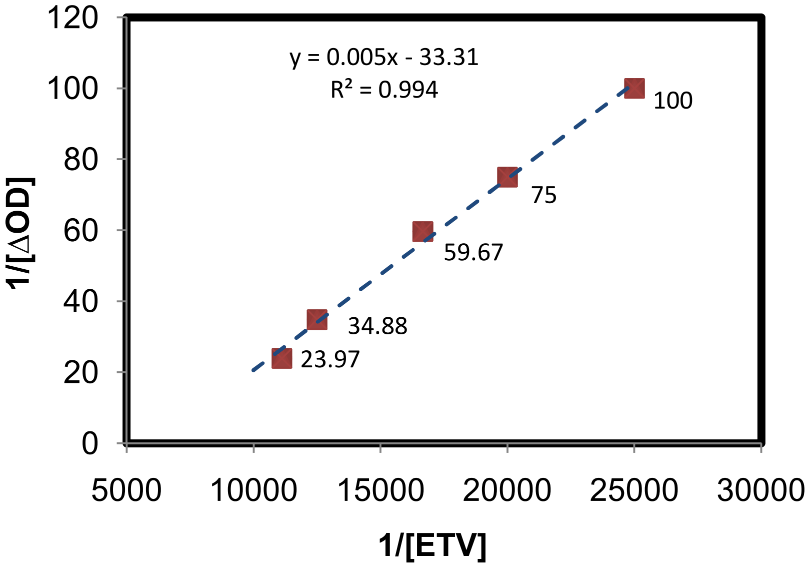

Fig S6. Benesi-Hildebrand plot of **ETV$^{3(+\bullet)}$ - CB[8]** titration by UV-visible spectrum.

Binding constant (K_a) value for complexed **ETV** was 6.2 X 10^3 M^{-1}

Viologen Star (ESV)

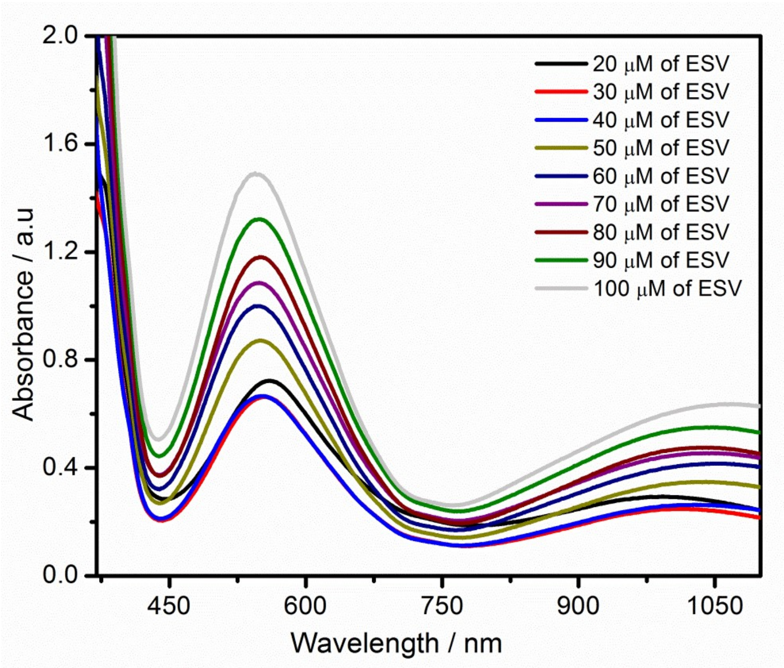

Fig S7. Absorption spectrum of **ESV$^{6(+\bullet)}$ - CB[8].**

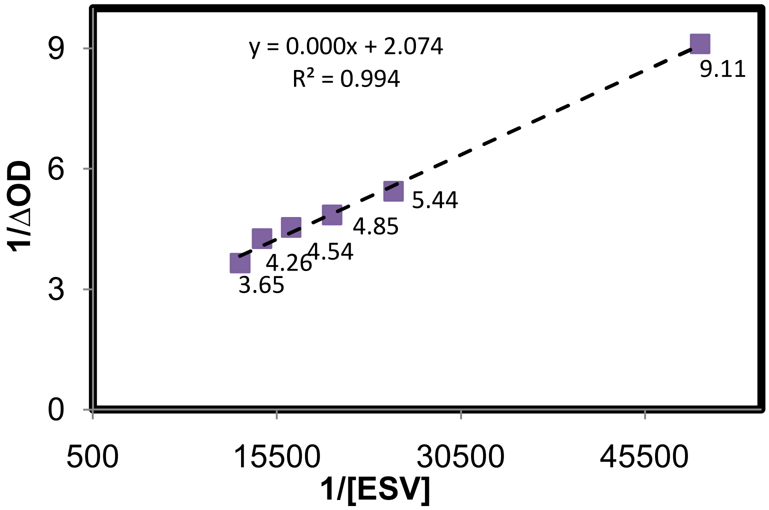

Fig S8. Benesi-Hildebrand plot of **ESV$^{6(+\bullet)}$ - CB[8]** titration by UV-visible spectrum.

Binding constant (K_a) value for complexed **ESV** was 2.1 X 10^4 M^{-1}

Zeta potential measurement

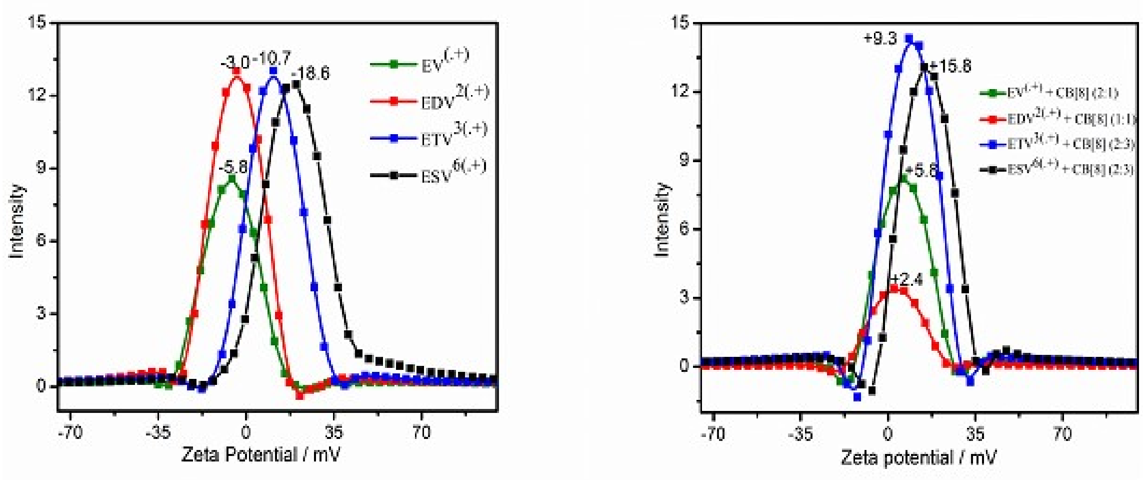

Fig S9. Zeta potential measurement of **EV**, **EDV**, **ETV** & **ESV** (0.2 mM) in aqueous solution containing excess of sodium dithionite in the a) absence and b) presence of **CB[8]** (0.1 mM).

HR-TEM

EDV + CB[8] + $Na_2S_2O_4$

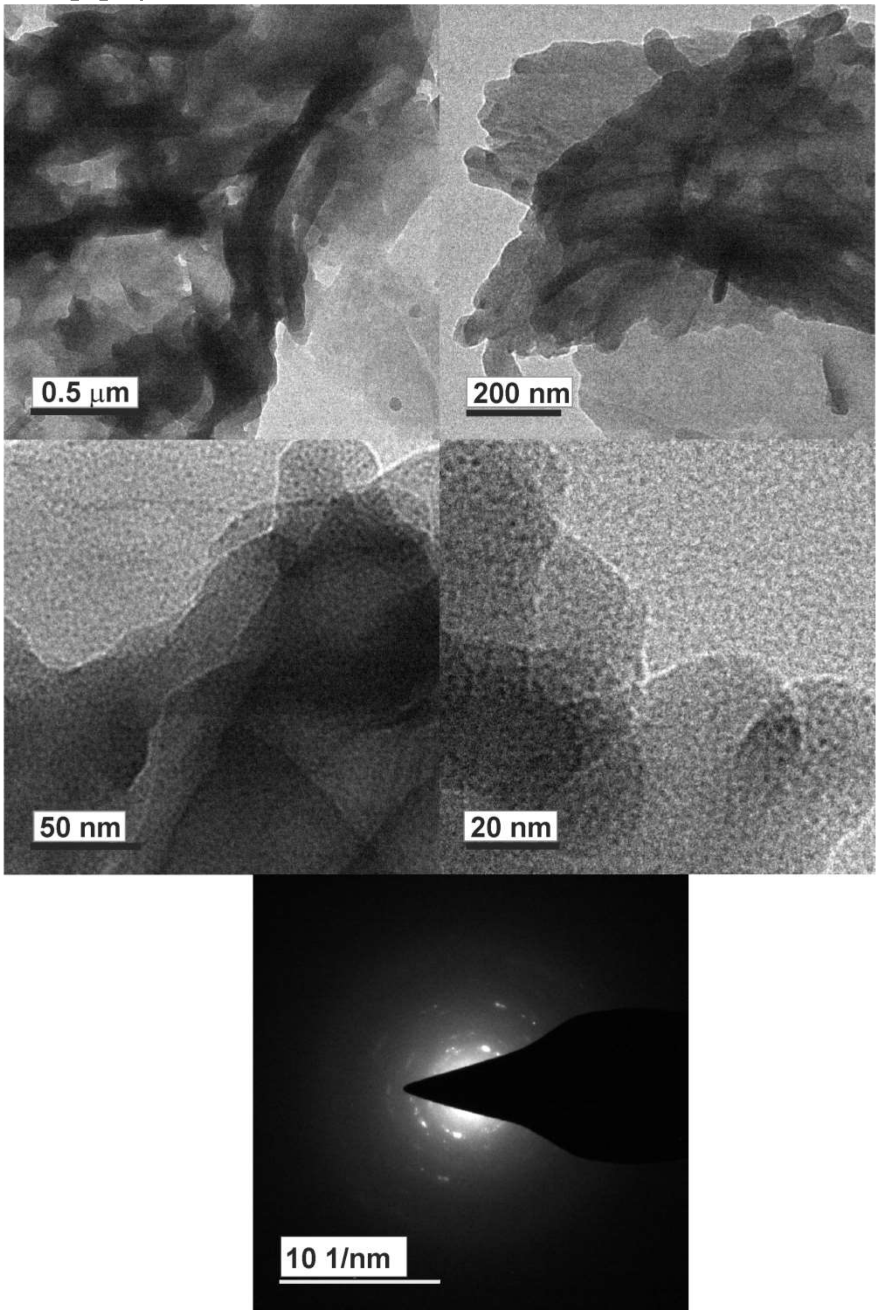

Figure S10. HR-TEM images of **EDV** (0.1 mM) **+ CB[8]** (2 equiv.) supramolecular polymer after reduction with sodium dithionite.

SAD (selected angle diffraction) pattern shows the semi crystalline nature of the supramolecular polymer.

ESV + CB[8] + $Na_2S_2O_4$

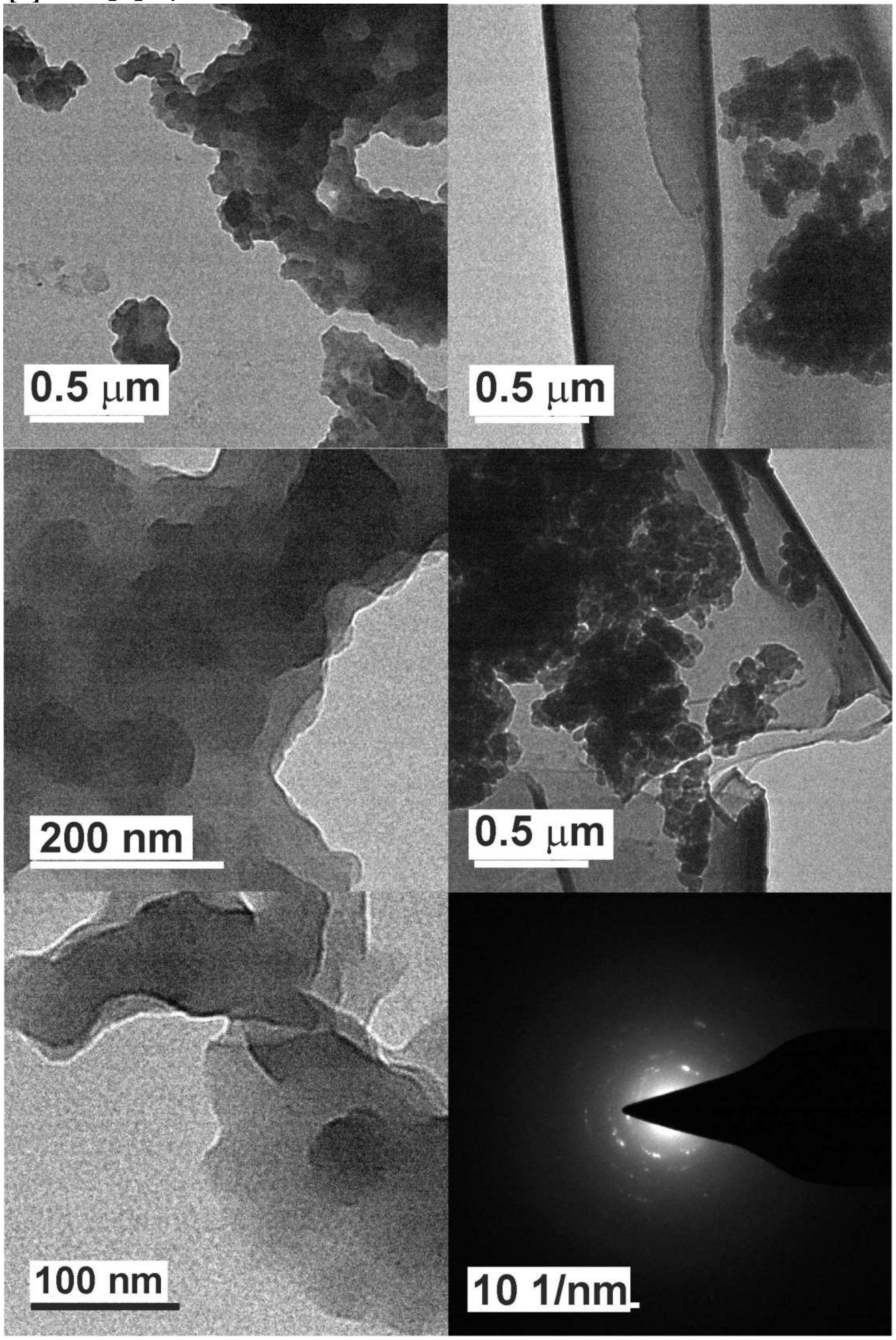

Figure S11. HR-TEM images of **ESV** (0.1 mM) **+ CB[8]** (6 equiv.) supramolecular polymer after reduction with sodium dithionite.

SAD pattern shows the semi-crystalline nature of the supramolecular polymer.

TEM

ETV + CB[8]

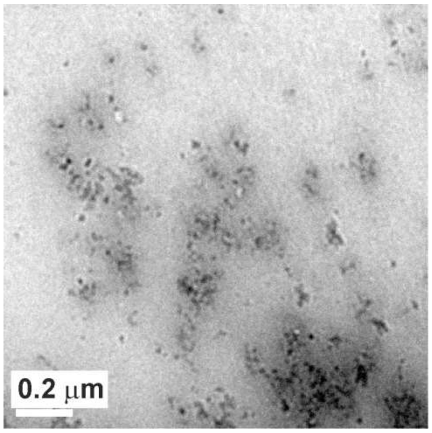

Figure S12. TEM image of **ETV** (0.1 mM) **+ CB[8]** (3 equiv.) before reduction with sodium dithionite.

ETV + CB[8] + $Na_2S_2O_4$

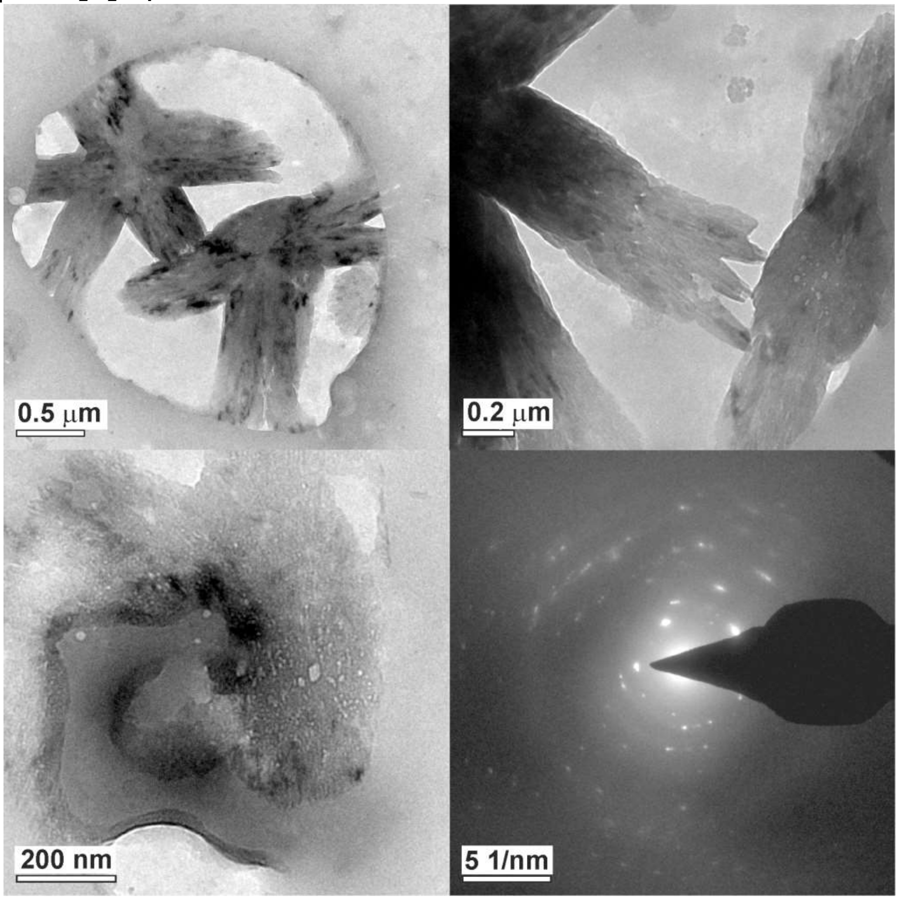

Figure S13. TEM images of **ETV** (0.1 mM) + **CB[8]** (3 equiv.) after reduction with sodium dithionite.
SAD pattern shows semi-crystalline nature of the supramolecular polymer.

SEM

The surface morphology of **EDV** and **ETV** samples were analyzed using SEM before and after reduction in the presence of CB[8]. Distinctive differences were observed in their morphology prior to reduction and after reduction with sodium dithionite. Noteworthy is that both the samples showed larger aggregates.

EDV+CB[8]

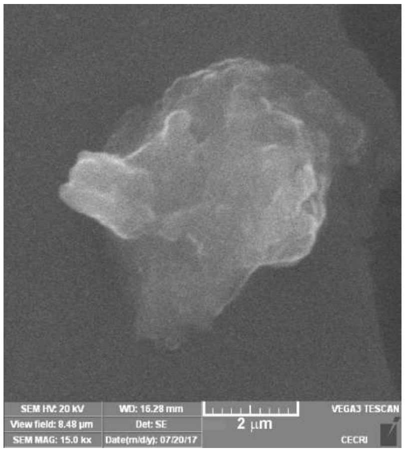

Figure S14. SEM image of **EDV** (0.1 mM) **+ CB[8]** (2 equiv.) before reduction with sodium dithionite.

EDV+CB[8]+$Na_2S_2O_4$

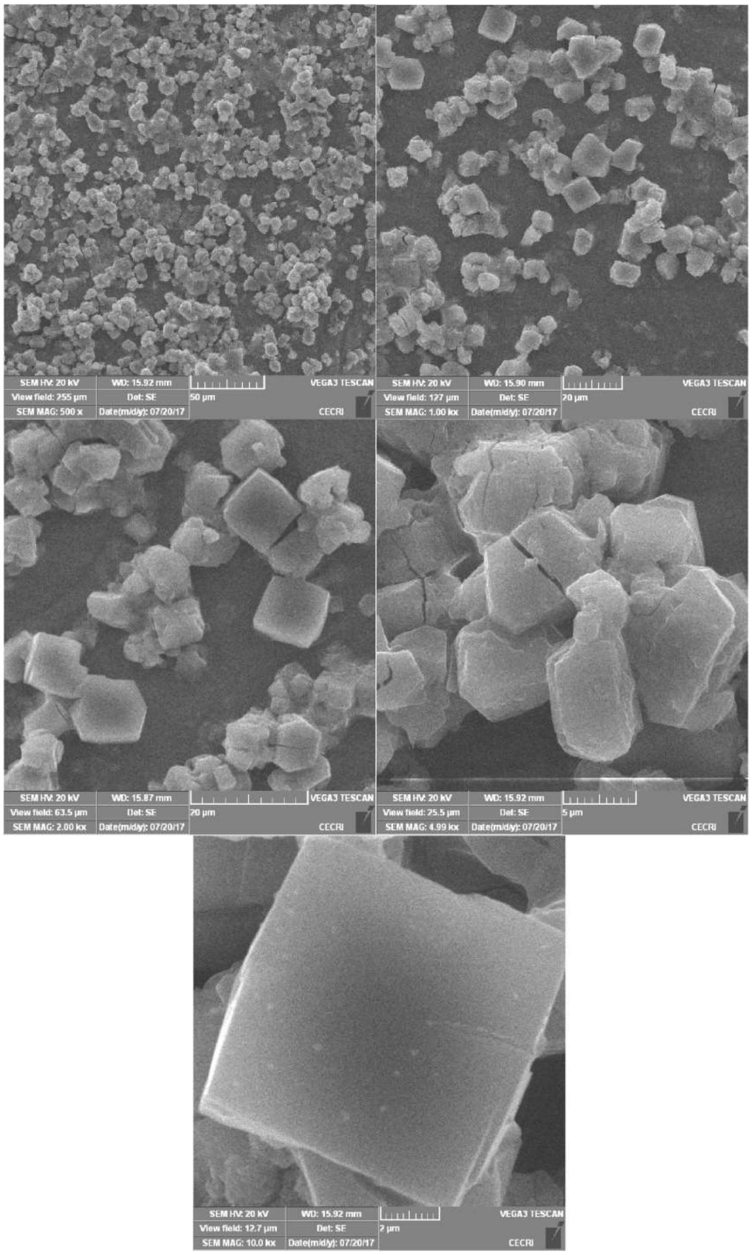

Figure S15. SEM images of **EDV** (0.1 mM) **+ CB[8]** (2 equiv.) after reduction with sodium dithionite.

ETV+CB[8]

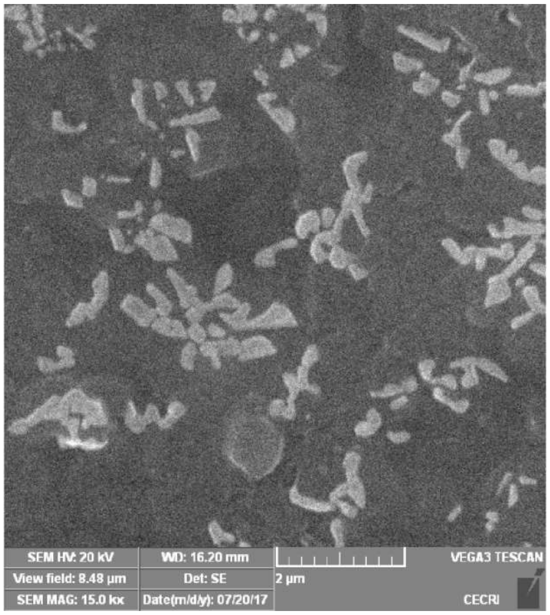

Figure S16. SEM image of **ETV** (0.1 mM) + **CB[8]** (3 equiv.) before reduction with sodium dithionite.

ETV+CB[8]+$Na_2S_2O_4$

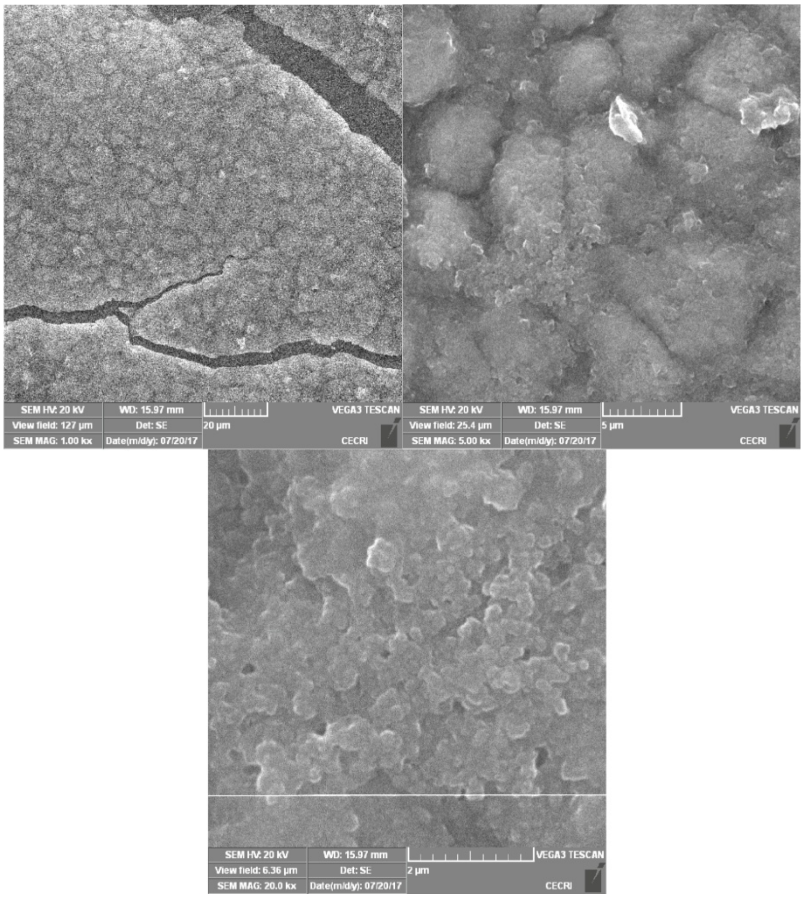

Figure S17. SEM images of **ETV** (0.1 mM) + **CB[8]** (3 equiv.) after reduction with sodium dithionite.

^{1}H, ^{13}C NMR spectrum of Host and Viologen-Guest molecules

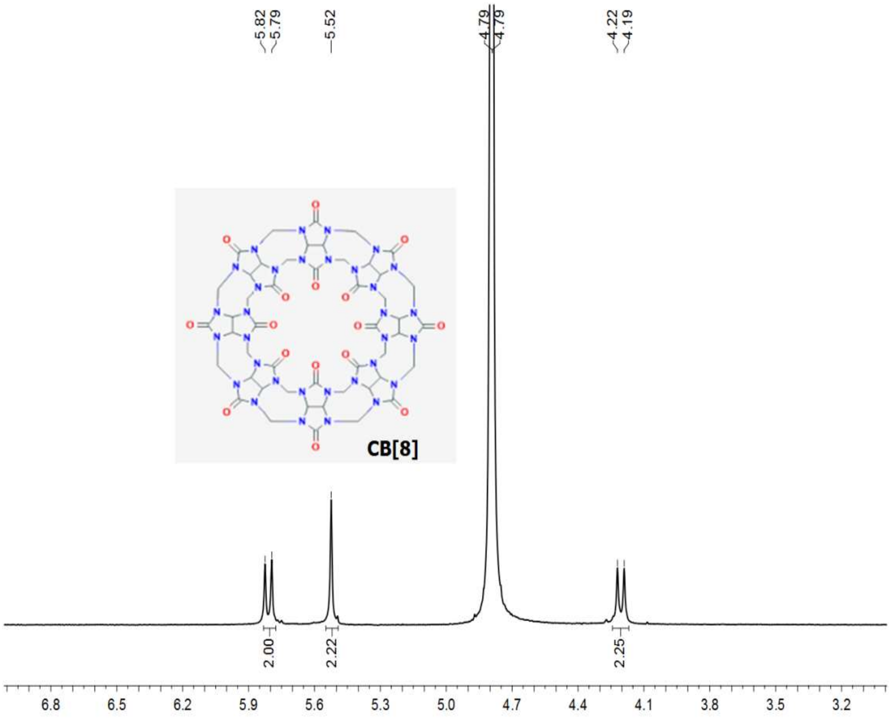

^{1}H NMR spectrum of **CB[8]**

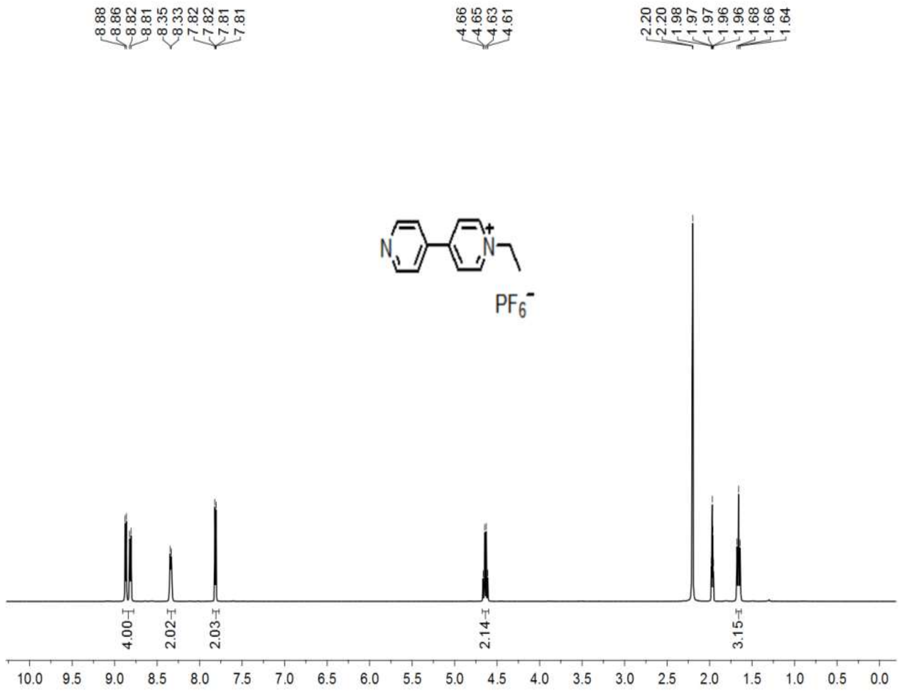

^{1}H NMR spectrum of **1** ^{13}C NMR spectrum of **1**

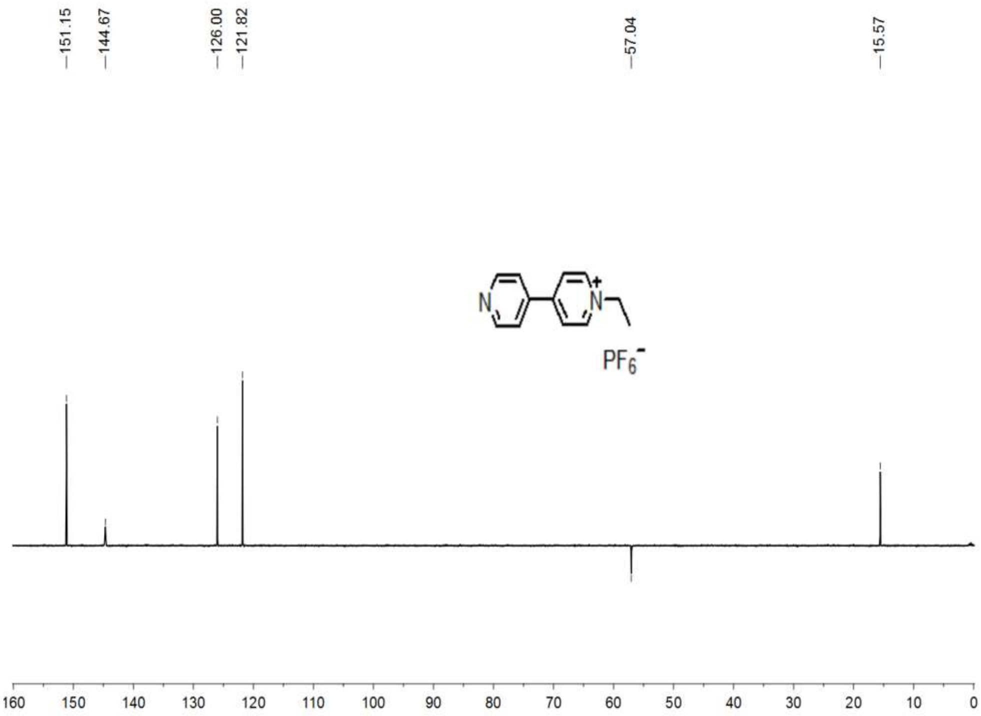

DEPT spectrum of **1**

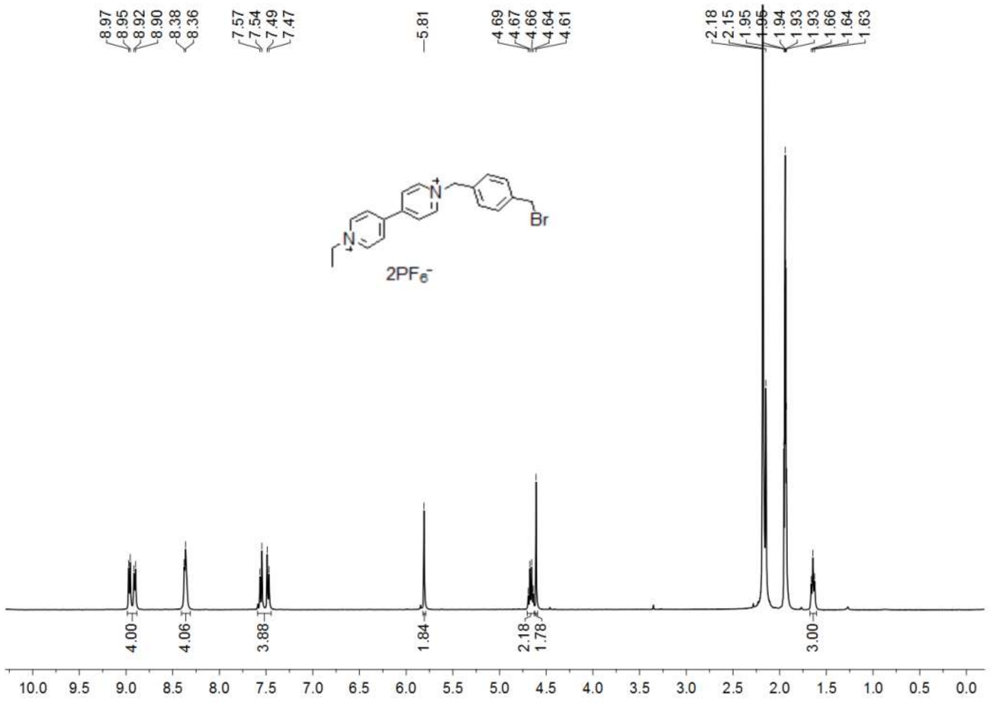

^{1}H NMR spectrum of **2**

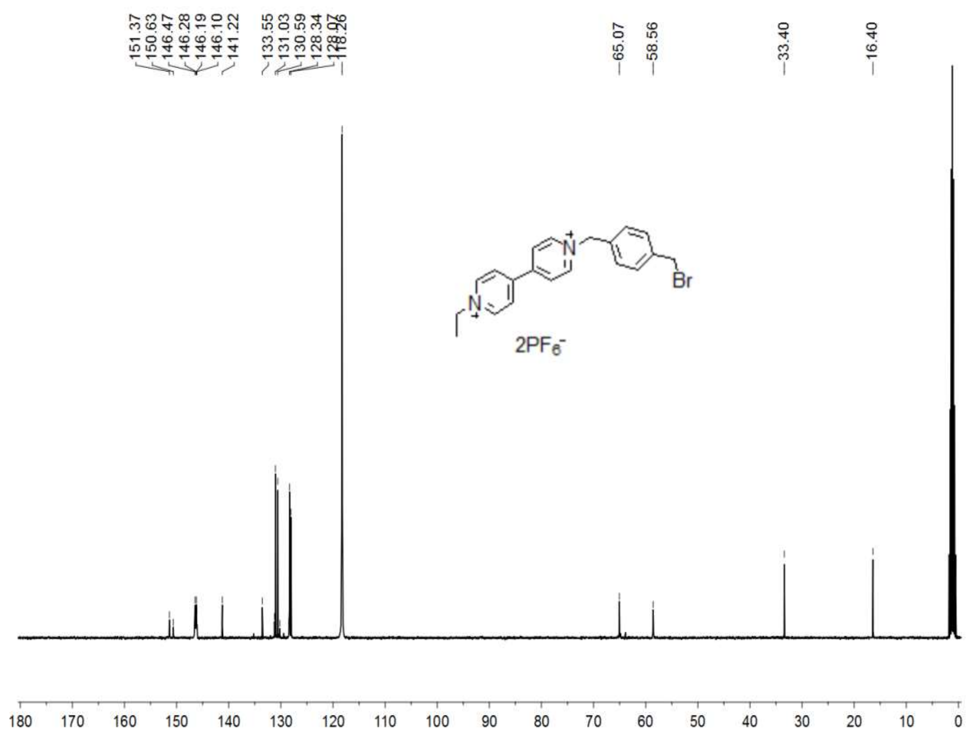

^{13}C NMR spectrum of **2**

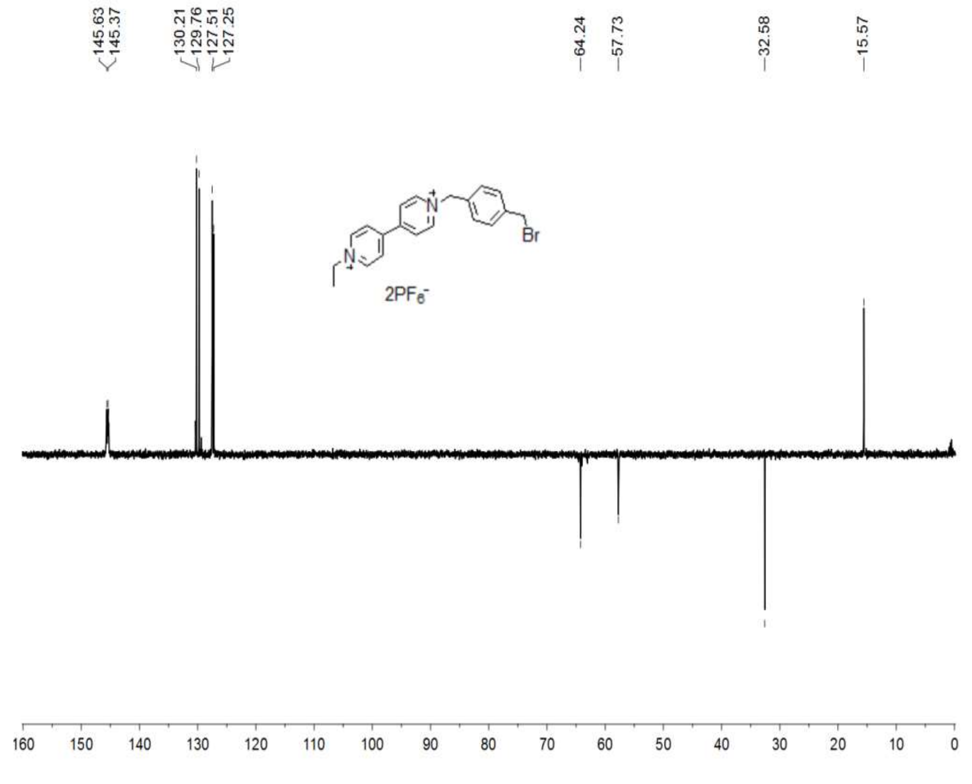

DEPT spectrum of **2**

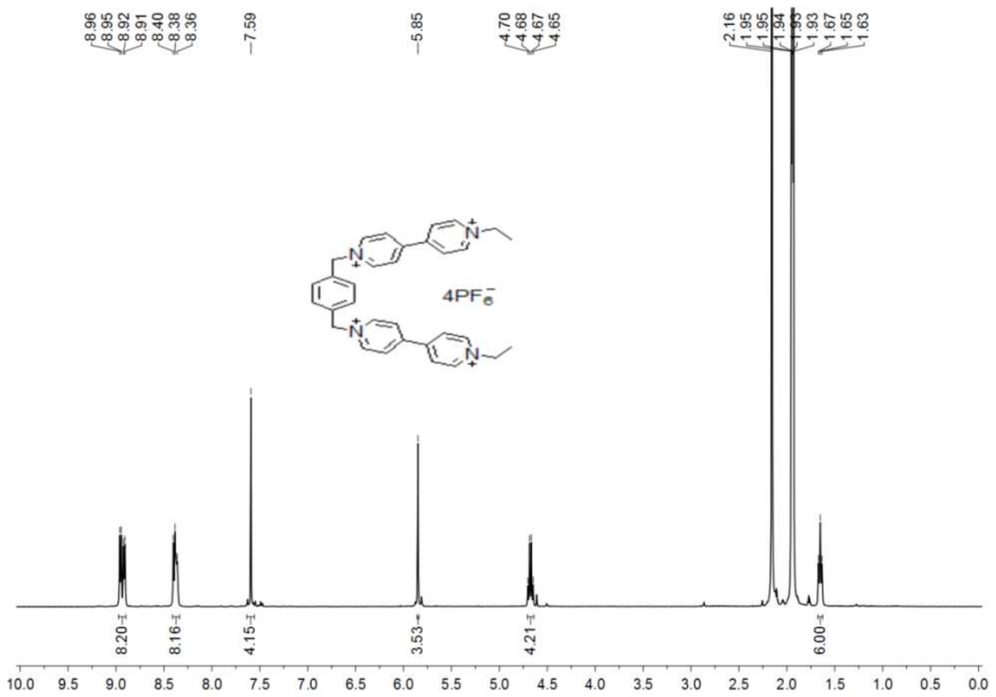

^{1}H NMR spectrum of **EDV**

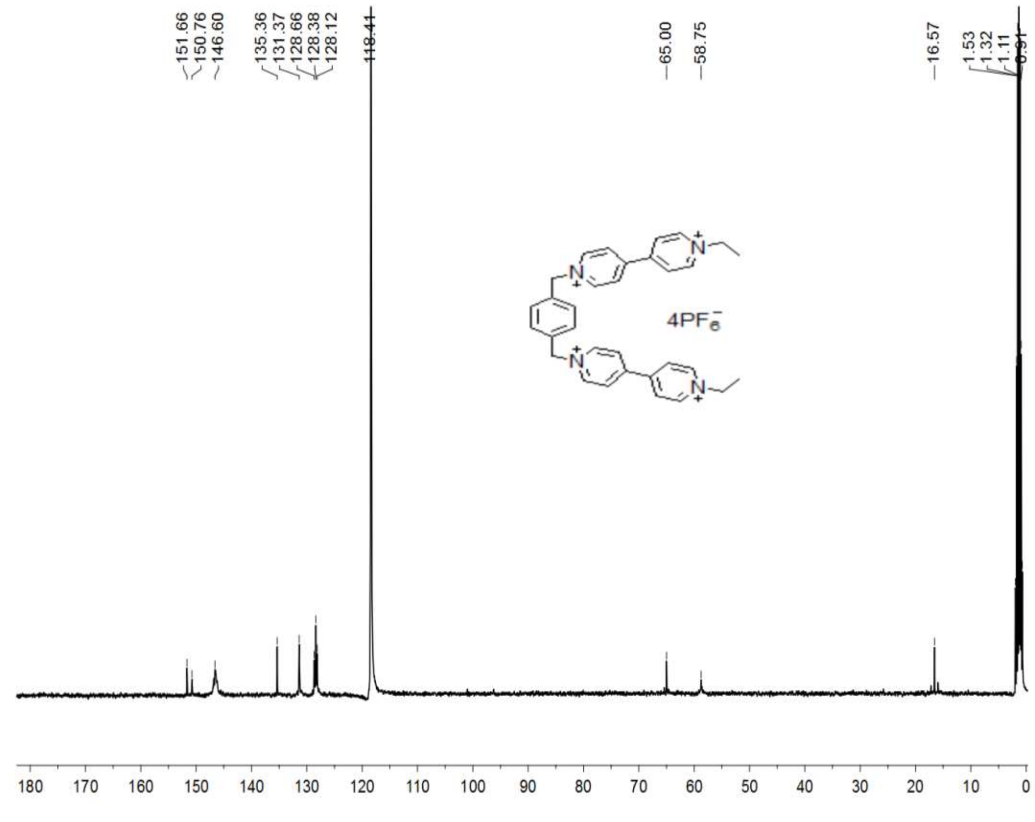

^{13}C NMR spectrum of **EDV**

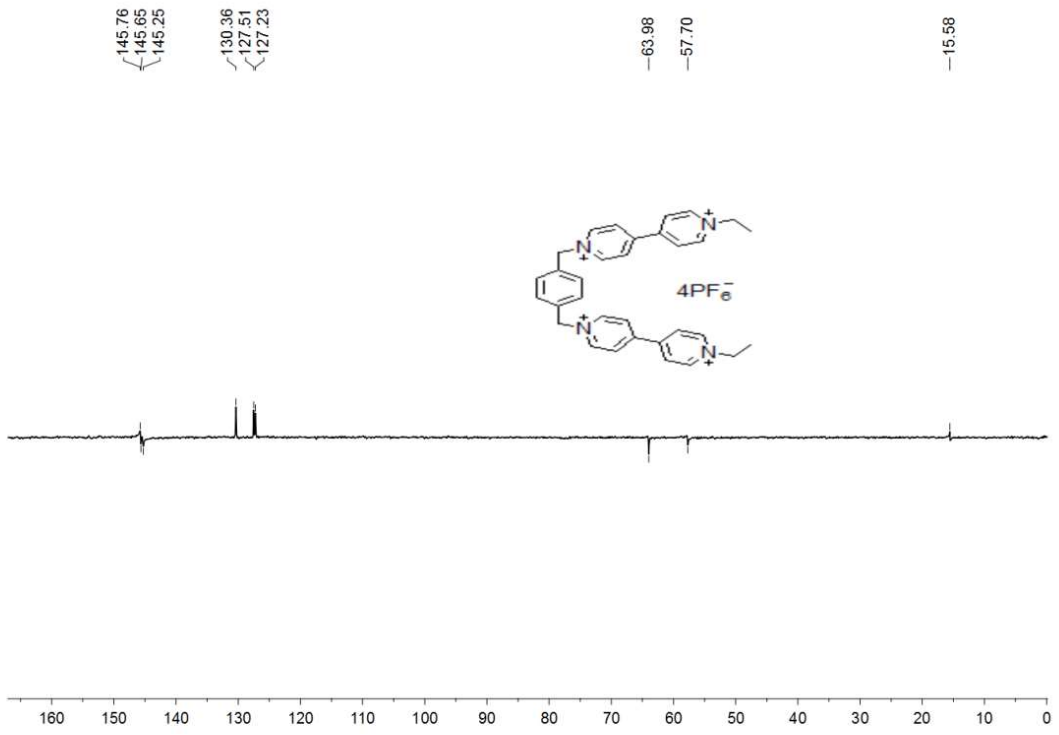

DEPT spectrum of **EDV**

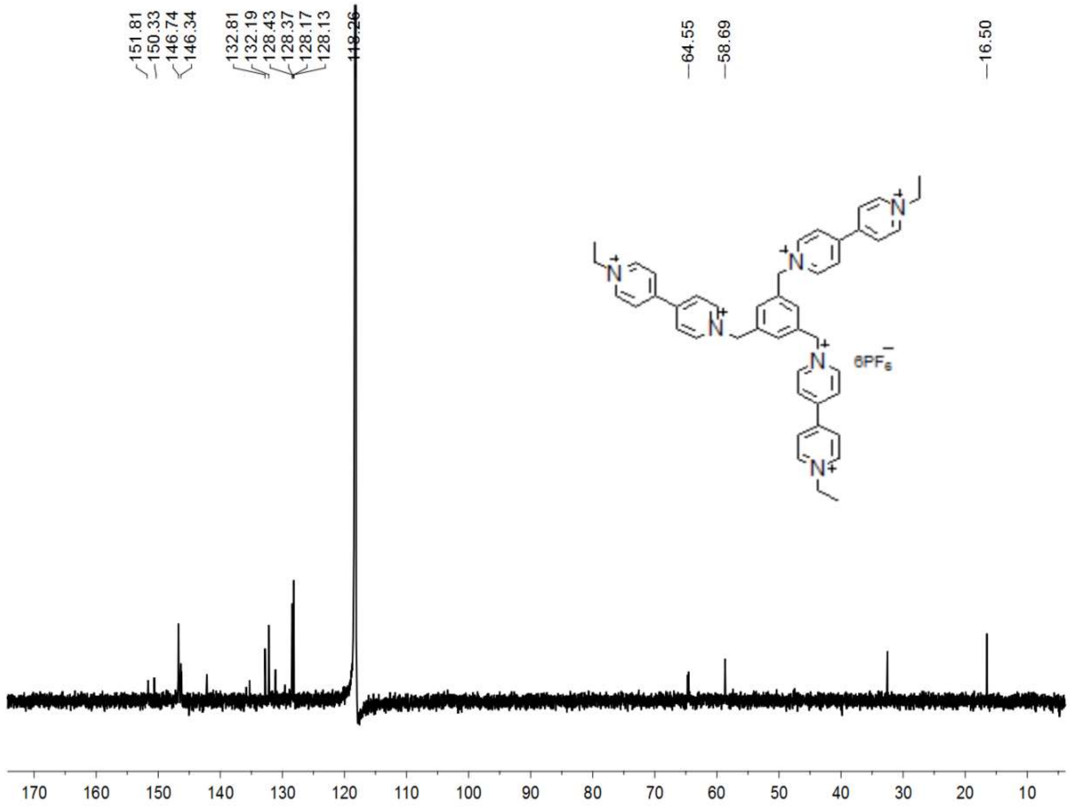

^{13}C NMR spectrum of **ETV**

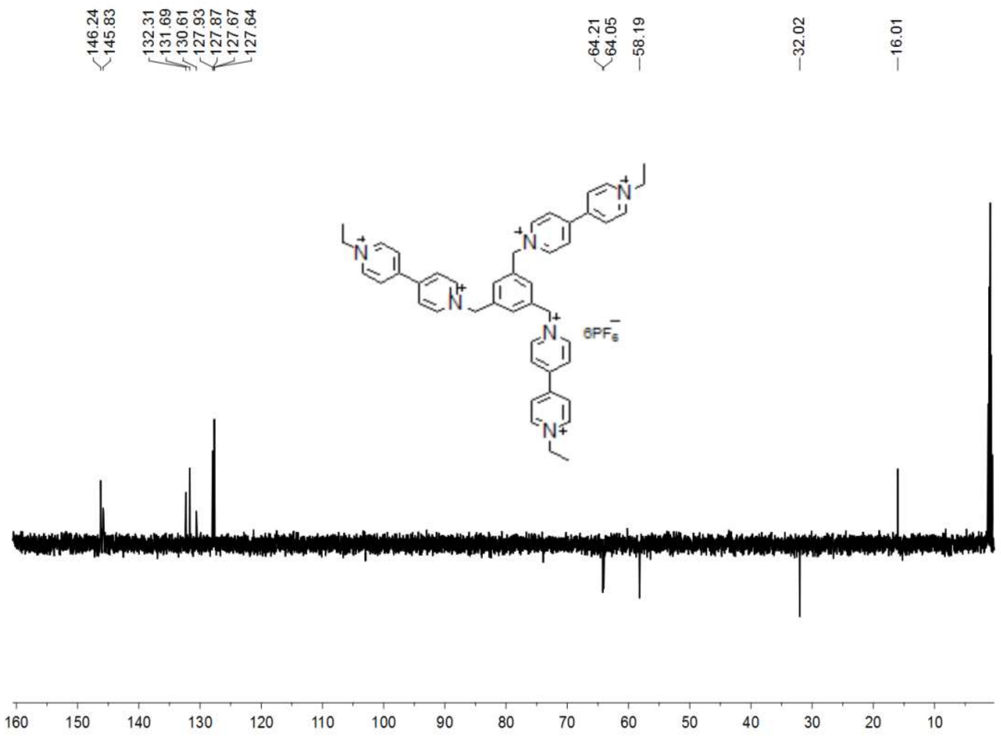

DEPT spectrum of **ETV**

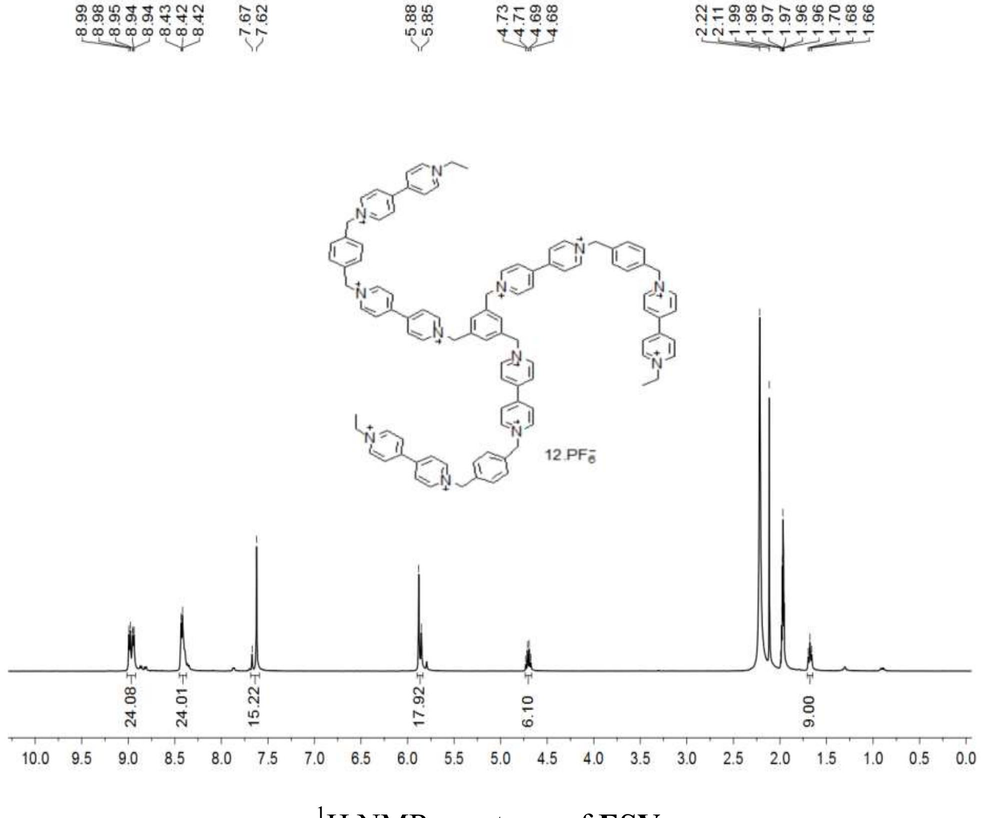

^{1}H NMR spectrum of **ESV**

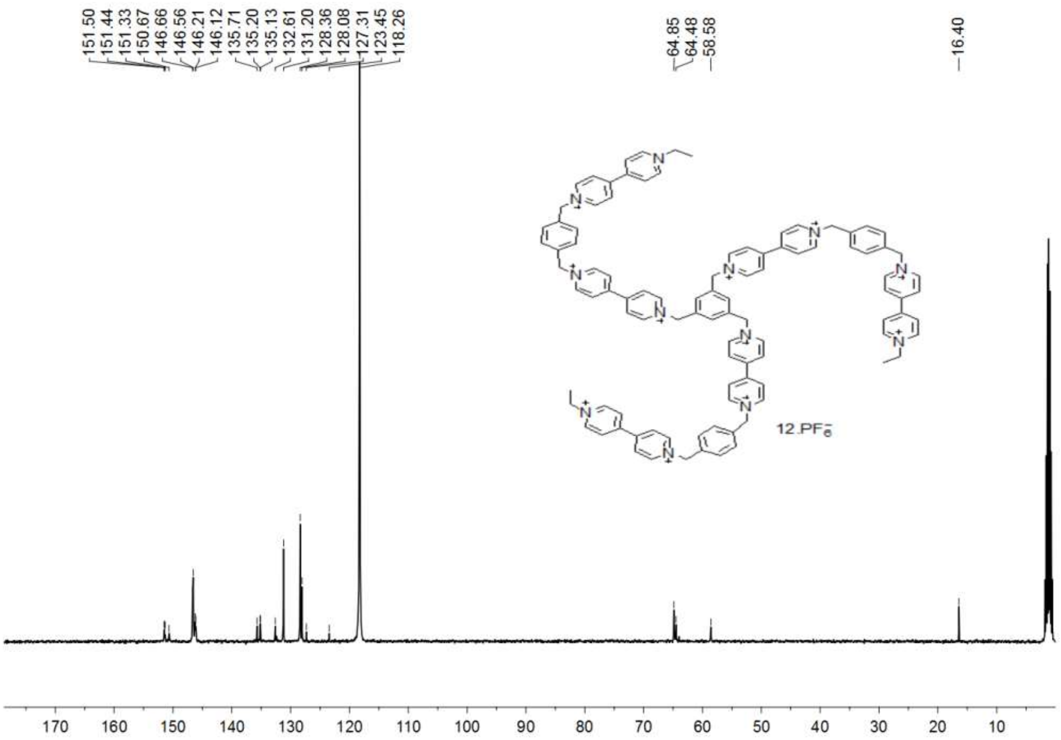

^{13}C NMR spectrum of **ESV**

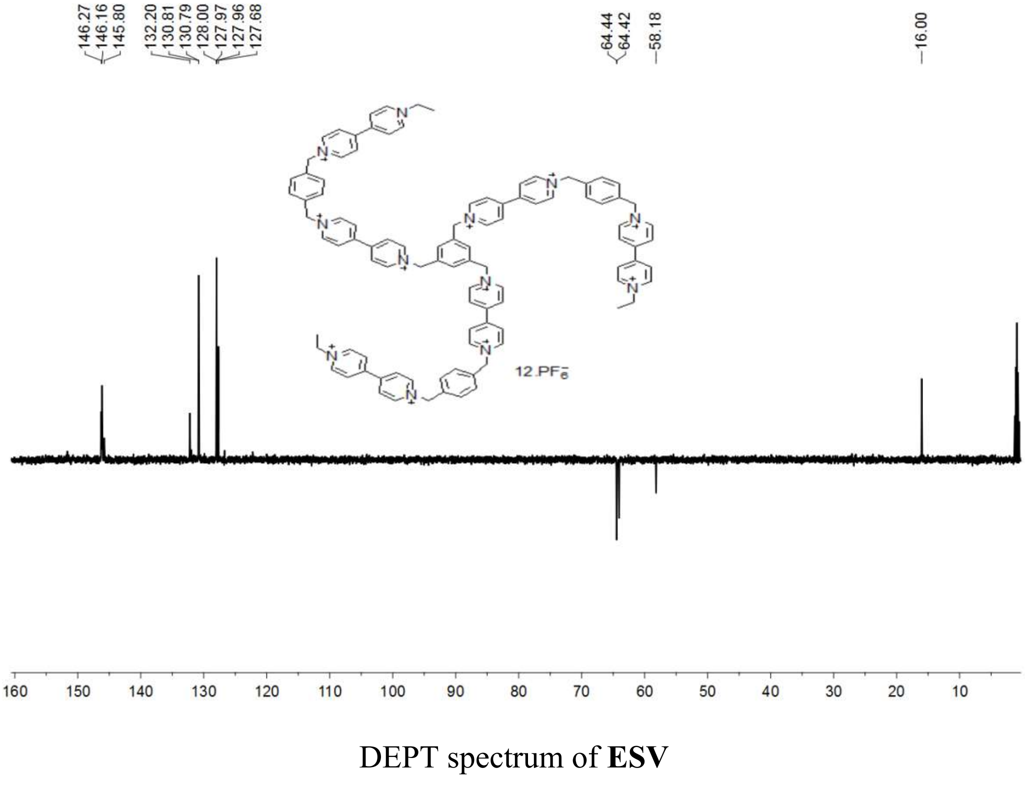

DEPT spectrum of **ESV**
